# Lifetime prevalence and adherence rate of cervical cancer screening among women living with HIV: a systematic review and meta‐analysis

**DOI:** 10.1002/jia2.26090

**Published:** 2023-05-29

**Authors:** Xiangrong Gao, Wanting Zhang, Jingyi Sun, Davies Adeloye, Huyi Jin, Igor Rudan, Peige Song, Mingjuan Jin

**Affiliations:** ^1^ Department of Public Health Second Affiliated Hospital Zhejiang University School of Medicine Hangzhou China; ^2^ Centre for Global Health Research Usher Institute of Population Health Sciences and Informatics University of Edinburgh Edinburgh UK

**Keywords:** women living with HIV, cervical cancer, screening, lifetime prevalence, adherence, systematic review

## Abstract

**Introduction:**

Women living with HIV (WLWH) are more likely to develop cervical cancer. Screening and available healthcare can effectively reduce its incidence and mortality rates. We aimed to summarize the lifetime prevalence and adherence rate of cervical cancer screening among WLWH across low‐ and middle‐income countries (LMICs), and high‐income countries (HICs).

**Methods:**

We systematically searched PubMed, Web of Science and Embase for studies published between database inception and 2 September 2022, without language or geographical restrictions. Those reporting the lifetime prevalence and/or adherence rate of cervical cancer screening among WLWH were included. Pooled estimates across LMICs and HICs were obtained using DerSimonian–Laird random‐effects models. When the number of eligible studies was greater than 10, we further conducted stratified analyses by the World Health Organization (WHO) region, setting (rural vs. urban), investigation year, screening method, type of cervical cancer screening programme, age and education level.

**Results:**

Among the 63 included articles, 26 provided data on lifetime prevalence, 24 on adherence rate and 13 on both. The pooled lifetime prevalence in LMICs was 30.2% (95% confidence interval [CI]: 21.0–41.3), compared to 92.4% in HICs (95% CI: 89.6–94.6). The pooled adherence rate was 20.1% in LMICs (95% CI: 16.4–24.3) and 59.5% in HICs (95% CI: 51.2–67.2).

**Discussion:**

There was a large gap in cervical cancer screening among WLWH between LMICs and HICs. Further analysis found that those in LMICs had higher lifetime prevalence in subgroups with urban settings, with older age and with higher education levels; and those in HICs had higher adherence in subgroups with younger age and with higher education levels.

**Conclusions:**

Cervical cancer screening among WLWH falls considerably short of the WHO's goal. There should be continuous efforts to further increase screening among these women, especially those residing in the rural areas of LMICs and with lower education levels.

## 1 INTRODUCTION

In 2019, there were 36.8 million people living with the human immunodeficiency virus (HIV) worldwide, of whom 20.1 million were women [1]. HIV infection causes immunodeficiency, which increases the risk of a wide range of infections and cancers [2]. Women living with HIV (WLWH) typically experience a greater likelihood of acquisition and persistence of the high‐risk human papillomavirus (HPV) and the progression of its downstream consequences, such as cervical cancer [3]. A recent meta‐analysis showed that WLWH have a six‐fold higher risk of developing cervical cancer than women without HIV [4].

The World Health Organization (WHO) has launched an ambitious call and global strategy to eliminate cervical cancer by 2030, with key measures of 90% HPV vaccination coverage for girls aged 9–14 years and 70% screening coverage for women aged 30–49 years [5]. While HPV vaccination is an effective long‐term modality for the primary prevention of cervical cancer, girls living with HIV have exhibited lower antibody titres than girls without HIV [6]. Screening has proven to be the most appropriate immediate strategy to reduce cervical cancer incidence and mortality by removing precancerous lesions and mortality by treating cancers at an early stage [7].

Despite the significance of screening, there is an obvious gap in cervical cancer screening between WLWH and women without HIV. For example, a follow‐up study from Denmark lasting 12 years suggested a worse adherence to cervical cancer screening among WLWH than among women without HIV [8]. Psychological and emotional barriers resulting from HIV‐related enacted and internalized stigmas, along with economic, social and healthcare system barriers, can contribute to this gap [9, 10]. Providing high levels of social support and developing more empathic person‐centred screening patterns have been identified as ways to promote an increase in cervical cancer screening among WLWH [11, 12]. Moreover, a clear understanding of the current screening prevalence and adherence is fundamental to guiding the implementation of a new screening programme. However, to date, relevant synthesized evidence for WLWH is absent.

To fill these gaps, we conducted a systematic review and meta‐analysis to estimate the lifetime prevalence and adherence to cervical cancer screening among WLWH across low‐ and middle‐income countries (LMICs) and high‐income countries (HICs), and to explore variations across different subgroups.

## 2 METHODS

This systematic review and meta‐analysis was conducted in accordance with the Preferred Reporting Items for Systematic Reviews and Meta‐analyses (PRISMA) guidelines, as well as the Meta‐analysis Of Observational Studies in Epidemiology (MOOSE) [13, 14]. The PRISMA checklist can be found in the Appendix of the Supplementary Materials. The study protocol was registered with PROSPERO (number CRD42021259593).

### 2.1 Search strategy

We systematically searched PubMed, Web of Science and Embase for studies published between database inception and 2 September 2022, without geographic or language restrictions. Search structures and keywords were tailored to each database. Two authors (XG and WZ) independently identified observational studies on the lifetime prevalence and/or adherence rate of cervical cancer screening among WLWH. The reference lists of the articles included were searched manually. The detailed search strings used for each database are shown in the Supplementary Materials (Table S1).

### 2.2 Study eligibility

For our systematic review, we selected studies that reported data on the lifetime prevalence and/or adherence rate of cervical cancer screening among WLWH. These data included sample size, number of subjects ever screened/adherent and/or lifetime prevalence/adherence rates. Studies were excluded if they were conducted in special WLWH (e.g. pregnant women) or were reported as reviews, conference abstracts, letters, editorials, commentaries or case series.

Duplicate records between databases were removed from our selection. After two authors (XG and JS) screened the titles and abstracts of the articles based on the inclusion and exclusion criteria, a full‐text review was conducted to determine their full eligibility. Disagreements during the review process were resolved through discussion with a third author (WZ) until a consensus was reached.

### 2.3 Outcome definition

Lifetime prevalence was defined as the proportion of WLWH who were ever screened for cervical cancer. Cervical cancer screening strategies and programmes were generally adopted and implemented by local governmental organizations after carefully considering its prevalence level; the availability of resources; and social, cultural and economic factors; hence, the recommended screening methods and intervals were not completely consistent across studies. Therefore, the adherence rate was defined as the proportion of WLWH who adhered to local guidelines for regular cervical cancer screening.

### 2.4 Quality assessment

To evaluate the risk of bias in the studies, we used a quality scale based on the Strengthening the Reporting of Observational Studies in Epidemiology (STROBE) statement with five domains (sample population, sample size, participation, outcome assessment and analytical methods) (Table S2) [15]. The total score, ranging from 0 to 10, represented the overall risk of bias for each article. Two authors (XG and HJ) independently assessed and scored each study according to the pre‐established criteria, and discrepancies were resolved through consultation with a third reviewer (WZ).

### 2.5 Data extraction

Two authors (XG and HJ) extracted the following data from all included articles: author, year published, year of investigation, country, geographic region (WHO/World Bank [WB] region), study setting (rural or urban), study name, screening method for the lifetime prevalence and/or screening guideline information (guideline name, definition of adherence, screening methods and intervals) for the adherence, sample size, the number of participants who had undergone cervical cancer screening and/or who were adherent to the screening guidelines, age (age range, mean/median age) of participants and education level (lower than high school [defined as illiteracy, primary school or middle school], high school [defined as high school or secondary school], higher than high school [defined as college, university, tertiary or above]). Following the geographic classification adopted by the WHO, the study regions were divided into the African Region (AFR), Region of the Americas (AMR), Southeast Asia Region (SEAR), European Region (EUR), Eastern Mediterranean Region (EMR) and Western Pacific Region (WPR). The study regions were also classified as LMICs and HICs based on their WB income categorization. Data on the type of national cervical screening programme (organized screening or opportunistic screening) were extracted from the WHO [16]. For articles in which the investigation year was not described, they were imputed by subtracting 3 and 5 years from the year of publication for lifetime prevalence and adherence rates, based on the mean time difference calculated from the available data (Tables S3 and S4).

### 2.6 Statistical analysis

The lifetime prevalence and adherence rates of cervical cancer screening in LMICs and HICs were pooled separately. For the adherence rate, only studies with a screening interval of 1 year were included in the meta‐analysis to reduce internal heterogeneity. The variance of the raw rate from each study was stabilized using logit transformation, and all estimates were presented afterwards. A random‐effects (DerSimonian–Laird method) meta‐analysis was used to calculate the combined estimates of the lifetime prevalence and adherence rates with 95% confidence intervals (CIs) [17, 18]. The heterogeneity of the data was tested through the Cochran *Q* test (*p* <0.05 represented significant heterogeneity) and the *I* ^2^ index (a value >50% may indicate substantial heterogeneity) [19, 20]. To examine whether single studies had a disproportionately excessive influence, a leave‐one‐out sensitivity analysis was conducted for each meta‐analysis [21]. Publication bias was detected qualitatively through the visual inspection of funnel plots and quantitatively through the Egger linear regression test and Begg's rank correlation test [22, 23, 24].

To investigate possible variations, in cases where the number of included studies was greater than 10, subgroup analyses were conducted according to its WHO region, setting, investigation year, type of national screening programme, screening method, age and education level. Since eligible data could be obtained from only five studies on lifetime prevalence in HICs and eight studies on adherence rates in LMICs, we limited the subgroup meta‐analysis for lifetime prevalence to LMICs and adherence rate to HICs.

All analyses were performed using R version 4.0.2 (R Foundation for Statistical Computing).

## 3 RESULTS

### 3.1 Literature review and study selection

**Figure 1:**
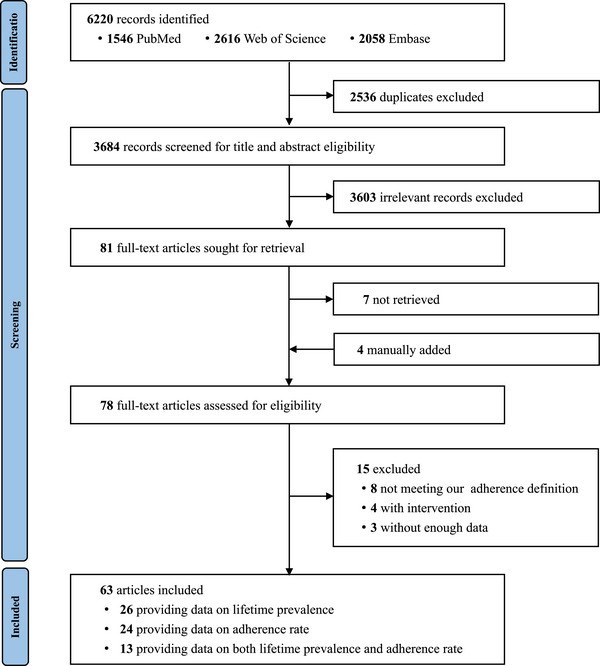
Study selection.

Overall, 6220 records were identified in the initial literature search. After removing duplicate records, initial screening of titles and abstracts, and manually adding four articles, 78 full texts were retrieved. Of these, 63 articles that met inclusion criteria were selected and 15 were excluded. Among the 63 articles, 26 provided data on lifetime prevalence, 24 on adherence rate and 13 on both (Figure 1 and Figure S1). There were 54 articles with a quality score of 6 or higher (Table S5).

### 3.2 Study characteristics

Among the 39 articles reporting the lifetime prevalence of cervical cancer screening (Table S6), 34 (87.2%) were conducted in LMICs, 28 (71.8%) were conducted in the AFR and 37 (94.9%) were published after 2010. For screening method, 16 (41.0%) used the Papanicolaou (Pap) test, 6 (15.4%) used Visual Inspection with Acetic Acid (VIA), 7 (17.9%) used Pap or VIA and 10 (25.6%) did not specify.

Among the 37 articles reporting adherence rates to cervical cancer screening (Table S7), 27 (73.0%) were conducted in HICs, 23 (62.2%) were conducted in AMR and 28 (75.7%) were published after 2010. For the screening method, 29 (78.4%) used the Pap test, 2 (5.4%) used VIA, 1 (2.7%) used Pap or VIA and 5 (13.5%) did not specify it. As for the screening interval, 29 (78.4%) designated 1‐year intervals, of which one provided data from six primary studies performed in different years [25]. For the other eight articles, 1, 3 and 2 designated 2‐, 3‐ and 5‐year intervals, respectively, while the two articles did not specify any concrete intervals (Figure S2).

### 3.3 Pooled lifetime prevalence and adherence rate of cervical cancer screening

**Figure 2:**
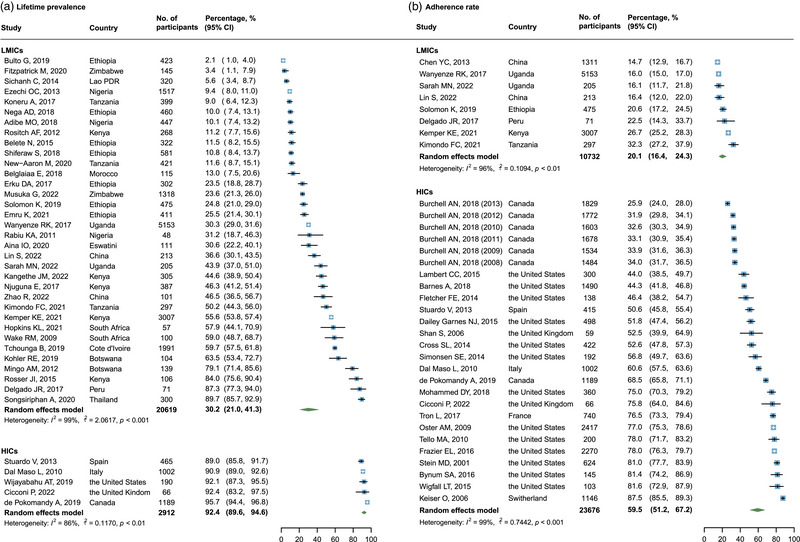
Pooled lifetime prevalence and adherence rate of cervical cancer screening.

Overall, 34 studies involving 20,619 WLWH in LMICs and 5 studies involving 2912 WLWH in HICs reported the lifetime prevalence of cervical cancer screening. The pooled estimates were 30.2% (95% CI: 21.0–41.3) in LMICs and 92.4% (95% CI: 89.6–94.6) in HICs (Figure 2). Leave‐one‐out sensitivity analysis showed that the pooled estimates were relatively robust, fluctuating between 28.6% (95% CI: 19.9–39.3) and 32.1% (95% CI: 22.8–43.0) in LMICs, and 90.6% (95% CI: 89.1–91.9) and 93.3% (95% CI: 90.4–95.3) in HICs (Figure S3). No obvious publication bias was found via the funnel plot, Egger test (*p* = 0.8855) or Begg's test (*p* = 0.4039) (Figure S4).

In total, eight studies involving 10,732 WLWH in LMICs and 26 studies involving 23,676 WLWH in HICs provided data on cervical cancer screening adherence. The pooled estimates were 20.1% (95% CI: 16.4–24.3) in LMICs and 59.5% (95% CI: 51.2–67.2) in HICs (Figure 2). Leave‐one‐out sensitivity analysis showed that the pooled estimates were relatively robust, between 18.6% (95% CI: 15.6–22.1) and 20.8% (95% CI: 16.7–25.7) in LMICs, and 57.9% (95% CI: 49.8–65.6) and 60.8% (95% CI: 52.8–68.4) in HICs (Figure S5). No obvious publication bias was found via the funnel plot, Egger test (*p* = 0.1455) or Begg's test (*p* = 0.3658) (Figure S6).

### 3.4 Subgroup lifetime prevalence of cervical cancer screening in LMICs

Table 1 presents the subgroup lifetime prevalence of cervical cancer screening in LMICs. The pooled estimates were 27.6% (95% CI: 18.9–38.2) in the AFR and 23.7 (95% CI: 7.6‐54.2) in the WPR. It was found to be higher in studies conducted in urban settings (30.3%, 95% CI: 14.8–52.0) than in those in rural settings (3.8%, 95% CI: 2.2–6.5). A higher estimate was also observed in studies based on organized screening programmes (45.4%, 95% CI: 23.0–69.7) than in studies based on opportunistic screening programmes (25.8%, 95% CI: 16.7–37.6). The pooled estimate was the highest among WLWH aged 45 years and older (39.2%, 95% CI: 21.5–60.3), followed by those 35–44 years old (35.2%, 95% CI: 23.6–49.0), and then by those younger than 35 years old (22.6%, 95% CI: 14.6–33.4). It was the highest among WLWH with education levels beyond high school (48.3%, 95% CI: 33.0–63.9), followed by those with up to a high school education (31.5%, 95% CI: 22.5–42.2), and then by those with education levels lower than high school (22.5%, 95% CI: 15.8–31.1).

**Table 1:** Subgroup analysis on the lifetime prevalence of cervical cancer screening in low‐ and middle‐income countries

Variable	No. of data points	No. of participants	No. of lifetime screening	Percentage (%) (95% CI)	Heterogeneity (*I* ^2^, %)	*p*‐Value of Q test	*p*‐Value between subgroups
**WHO region**							
Overall	34	20,619	6937	30.2 (21.0–41.3)	98.9	<0.0001	
AFR	28	19,499	6448	27.6 (18.9–38.2)	99.0	<0.0001	<0.0001
AMR	1	71	62	87.3 (77.4–93.3)	NA	NA	
EMR	1	115	15	13.0 (8.0–20.5)	NA	NA	
SEAR	1	300	269	89.7 (85.7–92.6)	NA	NA	
WPR	3	634	143	23.7 (7.6–54.2)	97.6	<0.0001	
**Setting**							
Overall	8	3526	1452	15.3 (6.2–33.1)	98.8	<0.0001	
Urban	5	3185	1439	30.3 (14.8–52.0)	99.1	<0.0001	<0.0001
Rural	3	341	13	3.8 (2.2–6.5)	0	0.8584	
**Investigation year**							
Overall	34	20,619	6937	30.2 (21.0–41.3)	98.9	<0.0001	
2010 and before	5	2072	357	33.3 (12.8–63.1)	98.9	<0.0001	0.9704
2011–2015	9	2574	605	29.3 (12.5–54.5)	98.2	<0.0001	
2016 and later	20	15,973	5975	29.9 (19.0–43.8)	99.0	<0.0001	
**Type of screening programme**							
Overall	31	19,502	6703	31.5 (21.5–43.5)	98.9	<0.0001	
Organized	10	2617	997	45.4 (23.0–69.7)	98.3	<0.0001	0.1411
Opportunistic	21	16,885	5706	25.8 (16.7–37.6)	99.1	<0.0001	
**Screening method**							
Overall	34	20,619	6937	30.2 (21.0–41.3)	98.9	<0.0001	
Pap	11	2010	661	34.6 (14.8–61.7)	98.5	<0.0001	0.9496
VIA	6	1719	513	31.1 (16.2–51.3)	98.1	<0.0001	
Pap/VIA	7	7605	3408	27.3 (11.6–51.7)	99.2	<0.0001	
Unspecified	10	9285	2355	27.2 (16.1–42.0)	98.1	<0.0001	
**Age (years)**							
Overall	38	13,317	5401	30.3 (22.4–39.6)	97.7	<0.0001	
Younger than 35	17	6049	1896	22.6 (14.6–33.4)	97.0	<0.0001	0.1754
35–44	8	4477	2235	35.2 (23.6–49.0)	98.1	<0.0001	
45 and older	13	2791	1270	39.2 (21.5–60.3)	97.0	<0.0001	
**Education level**							
Overall	41	13,213	5208	30.7 (24.3–38.0)	97.1	<0.0001	
Lower than high school	18	8186	2794	22.5 (15.8–31.1)	97.9	<0.0001	0.0124
High school	12	3083	1291	31.5 (22.5–42.2)	95.4	<0.0001	
Higher than high school	11	1944	1123	48.3 (33.0–63.9)	89.8	<0.0001	

Abbreviations: AFR, African Region; AMR, Region of the Americas; CI, confidence interval; EMR, Eastern Mediterranean Region; NA, not available; Pap, Papanicolaou; SEAR, South‐East Asia Region; VIA, Visual Inspection with Acetic Acid; WPR, Western Pacific Region.

### 3.5 Subgroup adherence rate of cervical cancer screening in HICs

Table 2 shows the subgroup adherence rates for cervical cancer screening in HICs. The pooled estimates were 56.4% (95% CI: 46.8–65.5) in the AMR and 69.3% (95% CI: 56.4–79.8) in the EUR. It was also observed to be consistent between studies based on organized screening programmes (59.0%, 95% CI: 49.8–67.6) and those based on opportunistic screening programmes (61.1%, 95% CI: 58.2–63.9). The estimate was the highest among WLWH younger than 35 years old (73.7%, 95% CI: 61.7–82.9), higher among those 35–44 years old (67.6%, 95% CI: 58.1–75.9) and the lowest among those 45 years and older (59.5%, 95% CI: 39.5–76.7). WLWH with education levels beyond high school (76.8%, 95% CI: 65.4–85.2) and those with up to a high school education (75.8%, 95% CI: 69.4–81.2) had higher estimates than those with education levels lower than high school (67.4%, 95% CI: 58.7–75.0).

**Table 2:** Subgroup analysis on the adherence rate of cervical cancer screening in high‐income countries

Variable	No. of data points	No. of participants	No. of adherence	Percentage (%) (95% CI)	Heterogeneity (*I* ^2^, %)	*p*‐Value of Q test	*p*‐Value between subgroups
**WHO region**							
Overall	26	23,676	12,634	59.5 (51.2–67.2)	99.3	<0.0001	
AMR	20	20,248	10,167	56.4 (46.8–65.5)	99.4	<0.0001	0.1072
EUR	6	3428	2467	69.3 (56.4–79.8)	98.3	<0.0001	
**Setting**							
Urban	4	2472	1308	63.4 (48.1–76.4)	98.0	<0.0001	‐
**Investigation year**							
Overall	26	23,676	12,634	59.5 (51.2–67.2)	99.3	<0.0001	
2010 and before	16	14,304	8476	58.8 (49.0–68.0)	99.3	<0.0001	0.8453
2011 and later	10	9372	4158	60.6 (45.8–73.6)	99.3	<0.0001	
**Screening method**							
Overall	26	23,676	12,634	59.5 (51.2–67.2)	99.3	<0.0001	
Pap	25	23,178	12,376	59.8 (51.2–67.8)	99.4	<0.0001	0.1038
Unspecified	1	498	258	51.8 (47.4–56.2)	NA	NA	
**Type of screening programme**							
Overall	26	23,676	12,634	59.5 (51.2–67.2)	99.3	<0.0001	
Organized	3	1127	688	59.0 (49.8–67.6)	99.4	0.0161	0.6607
Opportunistic	23	22,549	11,946	61.1 (58.2–63.9)	73.8	<0.0001	
**Age (years)**							
Overall	28	9443	6102	65.8 (55.9–74.5)	96.9	<0.0001	
Younger than 35	7	1606	1152	73.7 (61.7–82.9)	96.7	<0.0001	0.4194
35–44	8	3915	2572	67.6 (58.1–75.9)	97.8	<0.0001	
45 and older	13	3922	2378	59.5 (39.5–76.7)	96.6	<0.0001	
**Education level**							
Overall	20	8350	6208	72.7 (67.6–77.4)	92.0	<0.0001	
Lower than high school	8	2986	2129	67.4 (58.7–75.0)	93.7	<0.0001	0.1921
High school	8	4256	3216	75.8 (69.4–81.2)	91.8	<0.0001	
Higher than high school	4	1108	863	76.8 (65.4–85.2)	83.6	0.0004	

Abbreviations: AMR, WHO Region of the Americas; CI, confidence interval; EUR, WHO European Region; NA, not available; Pap, Papanicolaou.

## 4 DISCUSSION

This systematic review and meta‐analysis comprehensively described the lifetime prevalence and adherence rates of LMICs and HICs. Considerable inequity between LMICs and HICs was observed, with the lifetime prevalence of 30.2% and 92.4%, and adherence rates of 20.1% and 59.5%, respectively. Further subgroup analyses identified that the lifetime prevalence was higher among WLWH in urban settings, those living in areas with an organized screening programme, those who were older and those with higher levels of education; adherence was higher among those who were younger and those with higher levels of education.

Cervical cancer is a well‐known AIDS‐defining condition as WLWH have been proven to be at higher risk of its development and recurrence [4, 26, 27]. Accordingly, integrating cervical cancer screening into HIV/AIDS services has been developed to increase their uptake, which has shown to be an effective strategy as it facilitates the screening process and reduces health system expenditure [28, 29, 30]. Continuous attention, advocation and promotion by non‐governmental and governmental institutions contribute to better resource allocation and person‐centred strategies, eventually increasing the quality and uptake of cervical cancer prevention measures [12, 31].

HICs had a two‐fold higher lifetime prevalence than LMICs. This gap may be related to discrepancies in economic stability, health infrastructure, accessibility to screening services and life expectancy between persons in HICs and LMICs [32, 33, 34]. The differences across regions in the subgroup analysis of LMICs may not be globally representative, as approximately 85% of the included studies were from the AFR. WLWH residing in urban areas had a higher lifetime prevalence than those residing in rural areas, as urban areas are likely to have better economic and educational opportunities, and cultural support [35, 36]. As observed in studies involving women without HIV, systematic population‐based programmes can overcome financial and structural barriers to screening [37]. Therefore, the increasing trend in age‐related prevalence may be mainly due to cumulative effects.

HICs also showed approximately a two‐fold higher adherence rate than LMICs. Lower individual socio‐economic status and limited access to health services in LMICs are likely to contribute to poorer adherence rates [38]. In this research, we only included studies with 1‐year intervals in the meta‐analysis to obtain more homologous estimates since most international and national screening guidelines for WLWH adopted the Pap test as the primary screening method and VIA as an alternative screening method with 1‐year intervals during the eligible research period [39]. Nevertheless, the guidelines issued by WHO in 2021 recommended HPV DNA testing with 3‐ to 5‐year intervals as the primary choice; this is increasingly being used owing to its superior capacity for detecting precancerous and cancerous lesions [3, 5]. Adherence rates tend to decline with age, as older WLWH are more likely to have a lower perceived risk of cervical cancer [40, 41]. In studies conducted among women without HIV, an upward trend with education is reflected in the lifetime prevalence and adherence [42, 43]. Higher levels of education are usually associated with better health literacy, greater awareness of health risks and an improved access to health‐related resources [44, 45, 46].

This study possessed several strengths. It is the first systematic review and meta‐analysis on cervical cancer screening among WLWH measuring both lifetime prevalence and adherence rate. It also utilized a comprehensive search strategy, a double review process and stringent selection criteria. Furthermore, we conducted multiple subgroup analyses based on the evidence available on study characteristics, key elements of screening programmes and the socio‐demographic characteristics of participants; this facilitated a broader assessment of cervical cancer screening practices among WLWH.

However, this study also had limitations. Although we unified the definitions of lifetime prevalence and adherence before pooling the estimates, substantial heterogeneity among the studies included was detected. However, the heterogeneity may have been acceptable if pre‐defined inclusion criteria were met [47], and sensitivity analyses showed that the pooled estimates were robust. Additionally, further subgroup analyses were not performed for the lifetime prevalence in HICs and adherence rate in LMICs due to a lack of available data. Moreover, the estimates of the EMR, SEAR and WPR were not optimal due to insufficient evidence from these regions.

## 5 CONCLUSIONS

This study suggests that cervical cancer screening among WLWH falls considerably short of the WHO's goal and there are inequalities between LMICs and HICs. Furthermore, screening practices among WLWH in rural areas and those with lower education levels are particularly unsatisfactory. These data highlight the urgent need for continuous efforts to further increase screening. Additionally, more high‐quality epidemiological studies on cervical cancer screening and its determinants are needed.

## COMPETING INTERESTS

The authors declare that they have no competing interests.

## AUTHORS' CONTRIBUTIONS

MJ and PS planned the study and designed the methods. XG, WZ, JS and HJ contributed to the literature reviews and extracted data. XG, WZ, DA and IR did the statistical analyses. XG, WZ, PS and MJ wrote the first draft of the manuscript, with important contributions from IR and DA. MJ and PS accessed and verified the data. All authors interpreted the results, commented on drafts of the manuscript and approved the final version. All authors had full access to all the data in the study and had final responsibility for the decision to submit for publication.

## FUNDING

This work was supported by the National Key Research and Development Program of China (#2021YFC2500405). The funders had no role in study design, data collection and analysis, decision to publish or preparation of the manuscript.

## Supporting information

SUPPORTING INFORMATION
Additional information may be found under the Supporting Information tab for this article:
**Table S1**. Searching strategy
**Table S2**. Quality assessment scale for rating the risk of bias
**Table S3**. The time lag between investigation and publication in the included articles reporting the cervical cancer screening lifetime prevalence
**Table S4**. The time lag between investigation and publication in the included articles reporting the adherence to cervical cancer screening guidelines
**Table S5**. Quality scores for assessing the risk of bias for the included articles (*n* = 63)
**Table S6**. Basic characteristics of included studies reporting on cervical cancer screening lifetime prevalence (*n* = 39)
**Table S7**. Basic characteristics of included studies reporting on adherence to cervical cancer screening guidelines (*n* = 37)
**Figure S1**. Geographic distribution of the included studies
**Figure S2**. Adherence rates of cervical cancer screening with 2‐, 3‐, 5‐year interval and not specifying interval
**Figure S3**. Level‐one‐out sensitivity analysis of the influence of single study on the pooled cervical cancer screening lifetime prevalence
**Figure S4**. Publication bias of studies on the cervical cancer screening lifetime prevalence
**Figure S5**. Level‐one‐out sensitivity analysis of the influence of single study on the pooled cervical cancer screening adherence rate
**Figure S6**. Publication bias of studies on the cervical cancer screening adherence
**Appendix**. PRISMA checklist

## Data Availability

All data extracted for this systematic review are contained in the manuscript and supporting information.
