# Supplementary material for: Lifetime prevalence and adherence rate of cervical cancer screening among women living with HIV: a systematic review and meta‐analysis
Source: J Int AIDS Soc. 2023 May 29;26(5):e26090. doi: 10.1002/jia2.26090 (PMC10226725; doi:10.1002/jia2.26090)
Supplement: Supplementary file 1 — SUPPORTING INFORMATION Additional information may be found under the Supporting Information tab for this article: Table S1. Searching strategy Table S2. Quality assessment scale for rating the risk of bias Table S3. The time lag between investigation and publication in the included articles reporting the cervical cancer screening lifetime prevalence Table S4. The time lag between investigation and publication in the included articles reporting the adherence to cervical cancer screening guidelines Table S5. Quality scores for assessing the risk of bias for the included articles (n = 63) Table S6. Basic characteristics of included studies reporting on cervical cancer screening lifetime prevalence (n = 39) Table S7. Basic characteristics of included studies reporting on adherence to cervical cancer screening guidelines (n = 37) Figure S1. Geographic distribution of the included studies Figure S2. Adherence rates of cervical cancer screening with 2‐, 3‐, 5‐year interval and not specifying interval Figure S3. Level‐one‐out sensitivity analysis of the influence of single study on the pooled cervical cancer screening lifetime prevalence Figure S4. Publication bias of studies on the cervical cancer screening lifetime prevalence Figure S5. Level‐one‐out sensitivity analysis of the influence of single study on the pooled cervical cancer screening adherence rate Figure S6. Publication bias of studies on the cervical cancer screening adherence Appendix. PRISMA checklist [file JIA2-26-e26090-s001.pdf]

Lifetime prevalence and adherence rate of cervical cancer screening among women living with HIV: a systematic review and meta-analysis—Supplementary Materials

Xiangrong Gao, Wanting Zhang, Jingyi Sun, Davies Adeloye, Huyi Jin, Igor Rudan, Peige Song, Mingjuan Jin

Contents

Table S1. Searching strategy ··· 2

Table S2. Quality assessment scale for rating the risk of bias ··· 4

Table S3. The time lag between investigation and publication in the included articles reporting the cervical cancer screening lifetime prevalence ··· 5

Table S4. The time lag between investigation and publication in the included articles reporting the adherence to cervical cancer screening guidelines ··· 6

Table S5. Quality scores for assessing the risk of bias for the included articles (n=63) ··· 7

Table S6. Basic characteristics of included studies reporting on cervical cancer screening lifetime prevalence (n=39) ··· 9

Table S7. Basic characteristics of included studies reporting on adherence to cervical cancer screening guidelines (n=37) ··· 11

Figure S1. Geographic distribution of the included studies ··· 21

Figure S2. Adherence rates of cervical cancer screening with 2-year, 3-year, 5-year interval and not specifying interval ··· 22

Figure S3. Level-one-out sensitivity analysis of the influence of single study on the pooled cervical cancer screening lifetime prevalence ··· 23

Figure S4. Publication bias of studies on the cervical cancer screening lifetime prevalence ··· 24

Figure S5. Level-one-out sensitivity analysis of the influence of single study on the pooled cervical cancer screening adherence rate ··· 25

Figure S6. Publication bias of studies on the cervical cancer screening adherence ··· 26

Appendix. PRISMA checklist ··· 27

References ··· 30

Table S1. Search Strategy

Database	Access date	Search terms	No. of identified records
PubMed	2nd September, 2022	("cancer*"[Title/Abstract] OR "neoplas*"[Title/Abstract] OR "carcinoma*"[Title/Abstract] OR "tumor*"[Title/Abstract] OR "tumour*"[Title/Abstract]) AND ("cervi*"[Title/Abstract] OR "uterine neck"[Title/Abstract] OR "neck of uterus"[Title/Abstract]) AND ("screen*"[Title/Abstract] OR "early detection"[Title/Abstract] OR "papanicolaou test*"[Title/Abstract] OR "pap test*"[Title/Abstract] OR "pap smear*"[Title/Abstract] OR "vaginal smear*"[Title/Abstract] OR "hpv test*"[Title/Abstract] OR "cervical cytology"[Title/Abstract] OR "colposcop*"[Title/Abstract]) AND ("HIV"[Title/Abstract] OR "human immunedeficiency virus"[Title/Abstract] OR "human immune deficiency virus"[Title/Abstract] OR "human immunodeficiency virus"[Title/Abstract] OR "human immuno deficiency virus"[Title/Abstract] OR ("human immun*"[Title/Abstract] AND "deficiency virus"[Title/Abstract]) OR "acquired immunedeficiency syndrome"[Title/Abstract] OR "acquired immune deficiency syndrome"[Title/Abstract] OR "acquired immunodeficiency syndrome"[Title/Abstract] OR "acquired immuno deficiency syndrome"[Title/Abstract] OR ("acquired immun*"[Title/Abstract] AND "deficiency syndrome"[Title/Abstract]) OR "aids"[Title/Abstract])	1546
Embase	2nd September, 2022	(cancer*:ab,ti OR neoplas*:ab,ti OR carcinoma*:ab,ti OR tumor*:ab,ti OR tumour*:ab,ti) AND (cervi*:ab,ti OR 'uterine neck':ab,ti OR 'neck of uterus':ab,ti) AND (screen*:ab,ti OR 'early detection':ab,ti OR 'papanicolaou test*':ab,ti OR 'pap test*':ab,ti OR 'pap smear*':ab,ti OR 'vaginal smear*':ab,ti OR 'hpv test*':ab,ti OR 'cervical cytology':ab,ti OR colposcop*:ab,ti) AND (hiv:ab,ti OR 'human immunedeficiency virus':ab,ti OR 'human immune-deficiency virus':ab,ti OR 'human immunodeficiency virus':ab,ti OR 'human immuno-deficiency virus':ab,ti OR ('human immun*':ab,ti AND 'deficiency virus':ab,ti) OR 'acquired immunedeficiency syndrome':ab,ti OR 'acquired immune-deficiency syndrome':ab,ti OR 'acquired immunodeficiency syndrome':ab,ti OR 'acquired immuno-deficiency syndrome':ab,ti OR ('acquired immun*':ab,ti AND 'deficiency syndrome':ab,ti) OR aids*:ab,ti) AND ([embase]/lim OR [pubmed-not-medline]/lim)	2616

Web of Science	2nd September, 2022	#1：TS=(Cancer* OR neoplas* OR carcinoma* OR tumor* OR tumour*)<br>#2：TS=(cervi* OR uterine neck OR neck of uterus)<br>#3：TS=(screen* OR early detection OR Papanicolaou test* OR Pap test* OR Pap smear* OR vaginal smear* OR HPV test* OR cervical cytology OR colposcop*)<br>#4：TS=(HIV OR human immunedeficiency virus OR human immune-deficiency virus OR human immunodeficiency virus OR human immuno-deficiency virus OR ((human immun*) AND ("deficiency virus")) OR acquired immunedeficiency syndrome OR acquired immune-deficiency syndrome OR acquired immunodeficiency syndrome OR acquired immuno-deficiency syndrome OR ((acquired immun*) AND ("deficiency syndrome")) OR AIDS)<br>#5：#4 AND #3 AND #2 AND #1	2058

Table S2. Quality assessment scale for rating the risk of bias

Bias type	Low risk (score=2)	Moderate risk (score=1)	High risk (score=0)
Selection (sample population)	1) Sample from the general HIV-positive population, not a select group;<br>2) Consecutive unselected population;<br>3) Rationale for case and control selection explained.	1) Sample selected from large population but selection criteria not defined;<br>2) Sample selection ambiguous but may be representative;<br>3) Rationale for cases and controls not explained;<br>4) Eligibility criteria not explained;<br>5) Analysis to adjust for sampling strategy bias.	1) Highly select population making it difficult to generalise finding;<br>2) Sample selection ambiguous and sample unlikely to be representative.
Selection (sample size)	1) Sample size calculation performed and adequate.	1) Sample size calculation performed and reasons for not meeting sample size given;<br>2) Sample size calculation not performed but all eligible persons studied.	1) Sample size estimation unclear or only sub-sample studied.
Selection (participation rate)	1) High response rate (>85%).	1) Moderate response rate (70-85%).	1) Low response rate (<70%);<br>2) Response rate not reported.
Performance bias (outcome assessment)	1) Assessment from independent objective assessment or individual secure records;<br>2) Assessment from administrative database or register (hospital record or linkage record).	1) Assessment from self-report.	1) Assessment from non-validated data or generic estimate from the overall population.
Performance bias (analytical methods to control for bias)	1) Analysis appropriate for the type of sample (subgroup analysis/regression etc.).	1) Analysis does not account for common adjustment.	1) Data confusing.

Table S3. The time lag between investigation and publication in the included articles reporting the cervical cancer screening lifetime prevalence

Study	Year published	Year investigated	Time-lag (year)
Wake RM, 2009	2009	2006	3
Dal Maso L, 2010	2010	2007	3
Rabiu KA, 2011	2011	2009	2
Mingo AM, 2012	2012	2009	3
Rositch AF, 2012	2012	2008	4
Ezechi OC, 2013	2013	2004	9
Stuardo V, 2013	2013	2008	5
Sichanh C, 2014	2014	NA	NA
Belete N, 2015	2015	2014	1
Rosser JI, 2015	2015	2013	2
Delgado JR, 2017	2017	2014	3
Erku DA, 2017	2017	2017	0
Njuguna E, 2017	2017	2013	4
Wanyenze RK, 2017	2017	2016	1
Koneru A, 2017	2017	2012	5
Adibe MO, 2018	2018	NA	NA
Belglaiaa E, 2018	2018	2017	1
Nega AD, 2018	2018	2016	2
Shiferaw S, 2018	2018	2016	2
Bulto G, 2019	2019	2016	3
de Pokomandy A, 2019	2019	2014	5
Solomon K, 2019	2019	2018	1
Tchounga B, 2019	2019	2017	2
Wijayabahu AT, 2019	2019	2016	3
Kohler RE, 2019	2019	2017	2
Fitzpatrick M, 2020	2020	2017	3
New-Aaron M, 2020	2020	2017	3
Songsiriphan A, 2020	2020	2019	1
Aina IO, 2020	2020	2014	6
Emru K, 2021	2021	2015	6
Kemper KE, 2021	2021	2016	5
Kimondo FC, 2021	2021	2020	1
Hopkins KL, 2021	2021	2018	3
Musuka, G, 2022	2022	2016	6
Cicconi, P, 2022	2022	2020	2
Kangethe, J M, 2022	2022	2020	2
Sarah, Maria N, 2022	2022	2017	5
Zhao, R, 2022	2022	2019	3
Lin, S, 2022	2022	2019	3

Note: The average time-lag between investigation and publication was 3.11 years based on 37 studies with available data. NA=not available.

Table S4. The time lag between investigation and publication in the included articles reporting the adherence to cervical cancer screening guidelines

Study	Year published	Year investigated	Time-lag (year)
Stein MD, 2001	2001	1996	5
Keiser O, 2006	2006	2003	3
Shah S, 2006	2006	2003	3
Oster AM, 2009	2009	2002	7
Dal Maso L, 2010	2010	2007	3
Leece P, 2010	2010	2004	6
Logan JL, 2010	2010	2003	7
Rahangdale L, 2010	2010	2004	6
Tello MA, 2010	2010	2008	2
Stuardo V, 2013	2013	2008	5
Chen YC, 2013	2013	2005	8
Cross SL, 2014	2014	2010	4
Fletcher FE, 2014	2014	2008	6
Simonsen SE, 2014	2014	2009	5
Dailey Garnes NJ, 2015	2015	2007	8
Lambert CC, 2015	2015	NA	NA
Wigfall LT, 2015	2015	2012	3
Bynum SA, 2016	2016	2011	5
Frazier EL, 2016	2016	2010	6
Ogunwale AN, 2016	2016	2012	4
Delgado JR, 2017	2017	2014	3
Tron L, 2017	2017	2011	6
Wanyenze RK, 2017	2017	2016	1
Barnes A, 2018	2018	2012	6
Burchell AN, 2018 (2008)	2018	2008	10
Burchell AN, 2018 (2009)	2018	2009	9
Burchell AN, 2018 (2010)	2018	2010	8
Burchell AN, 2018 (2011)	2018	2011	7
Burchell AN, 2018 (2012)	2018	2012	6
Burchell AN, 2018 (2013)	2018	2013	5
Mohammed DY, 2018	2018	2016	2
Assefa AA, 2019	2019	2019	0
de Pokomandy A, 2019	2019	2014	5
Short W R, 2019	2019	2014	5
Solomon K, 2019	2019	2018	1
Wijayabahu AT, 2019	2019	2016	3
Songsiriphan A, 2020	2020	2019	1
Kemper KE, 2021	2021	2016	5
Kimondo FC, 2021	2021	2020	1
Cicconi, P, 2022	2022	2020	2
Sarah, Maria N, 2022	2022	2017	5
Lin, S, 2022	2022	2019	3

Note: The average time-lag between investigation and publication was 4·63 years based on 41 studies with available data. NA=not available.

Table S5. Quality scores for assessing the risk of bias for the included articles (n=63)

Study	Quality score					
	Sample population	Sample size	Participation	Outcome assessment	Analytical methods	Total scores
Stein MD, 2001	1	1	2	1	1	6
Keiser O, 2006	2	1	2	1	1	7
Shah S, 2006	2	1	1	2	1	7
Oster AM, 2009	2	1	1	1	2	7
Wake RM, 2009	2	1	2	1	1	7
Dal Maso L, 2010	1	1	2	1	2	7
Leece P, 2010	1	1	2	2	1	7
Logan JL, 2010	2	1	2	2	1	8
Rahangdale L, 2010	1	1	2	2	1	7
Tello MA, 2010	2	1	2	2	2	9
Rabiu KA, 2011	1	2	2	1	1	7
Mingo AM, 2012	1	1	2	1	1	6
Rositch AF, 2012	1	1	1	1	1	5
Ezechi OC, 2013	1	2	2	1	2	8
Stuardo V, 2013	1	1	2	1	1	6
Chen YC, 2013	2	1	1	2	1	7
Cross SL, 2014	1	1	2	2	1	7
Fletcher FE, 2014	1	1	2	2	2	8
Sichanh C, 2014	2	1	2	1	1	7
Simonsen SE, 2014	2	1	2	2	1	8
Belete N, 2015	1	2	2	1	2	8
Dailey Garnes NJ, 2015	1	1	2	2	2	8
Lambert CC, 2015	2	2	2	1	1	8
Rosser JI, 2015	2	1	2	1	2	8
Wigfall LT, 2015	2	1	2	1	1	7
Bynum SA, 2016	2	1	2	1	2	8
Frazier EL, 2016	2	1	1	1	2	7
Ogunwale AN, 2016	2	1	2	1	2	8
Delgado JR, 2017	2	1	2	1	1	7
Erku DA, 2017	2	2	2	1	2	9
Njuguna E, 2017	2	1	2	1	1	7
Tron L, 2017	2	1	2	1	2	8
Wanyenze RK, 2017	2	2	2	1	2	9
Koneru A, 2017	2	2	1	1	2	8
Adibe MO, 2018	1	2	2	1	1	7
Barnes A, 2018	2	1	2	2	2	9
Belglaiaa E, 2018	0	1	2	1	1	5
Burchell AN, 2018	1	1	0	2	1	5
Nega AD, 2018	1	2	2	1	2	8

Study	Quality score					
	Sample population	Sample size	Participation	Outcome assessment	Analytical methods	Total scores
Shiferaw S, 2018	1	2	2	1	1	7
Mohammed DY, 2018	2	1	1	2	2	8
Assefa AA, 2019	1	2	2	1	2	8
Bulto G, 2019	1	2	2	1	1	7
de Pokomandy A, 2019	2	1	1	1	1	6
Short W R, 2019	2	1	0	2	2	7
Solomon K, 2019	2	2	2	1	2	9
Tchounga B, 2019	1	1	2	1	2	7
Wijayabahu AT, 2019	1	1	0	1	1	4
Kohler RE, 2019	2	1	1	1	1	6
Fitzpatrick M, 2020	2	0	1	1	1	5
New-Aaron M, 2020	2	0	2	1	1	6
Songsiriphan A, 2020	2	2	0	1	0	5
Aina IO, 2020	1	1	1	1	1	5
Emru K, 2021	1	2	2	1	2	8
Kemper KE, 2021	2	1	0	1	2	6
Kimondo FC, 2021	2	2	2	1	1	8
Hopkins KL, 2021	1	1	1	1	1	5
Musuka, G, 2022	1	1	1	1	1	5
Cicconi, P, 2022	2	1	2	1	1	7
Kangethe, J M, 2022	2	1	1	1	2	7
Sarah, Maria N, 2022	2	2	1	1	1	7
Zhao, R, 2022	2	1	1	1	1	6
Lin, S, 2022	2	2	1	1	2	8

Table S6 Basic characteristics of included studies reporting cervical cancer screening lifetime prevalence (n=39)

Study	Year investigated	Country	WHO region	WB region	Study setting	Study name	Type of cervical cancer screening programme	Screening method	No. of participants [a]	No. of lifetime screening	Age range (years)
Wake RM, 2009 [1]	2006	South Africa	AFR	LMIC	Urban	NA	Opportunistic	Pap	100	59	21–64
Dal Maso L, 2010 [2]	2007	Italy	EUR	HIC	NA	NA	Organised population-based	Pap	1002	911	NA
Rabiu KA, 2011 [3]	2009	Nigeria	AFR	LMIC	NA	NA	Opportunistic	Pap	48	15	17–60
Mingo AM, 2012 [4]	2009	Botswana	AFR	LMIC	NA	NA	Organised population-based	Pap	139	110	20–84
Rositch AF, 2012 [5]	2008	Kenya	AFR	LMIC	NA	NA	Opportunistic	Pap	268	30	NA
Ezechi OC, 2013 [6]	2004	Nigeria	AFR	LMIC	NA	NA	Opportunistic	NA	1517	143	18–57
Stuardo V, 2013 [7]	2008	Spain	EUR	HIC	NA	PISCIS	Opportunistic	Pap	465	414	NA
Sichanh C, 2014 [8]	2011	Lao PDR	WPR	LMIC	NA	NA	Opportunistic	Pap	320	18	25–65
Belete N, 2015 [9]	2014	Ethiopia	AFR	LMIC	NA	NA	Opportunistic	NA	322	37	18–69
Rosser JI, 2015 [10]	2013	Kenya	AFR	LMIC	NA	NA	Opportunistic	NA	106	89	23–64
Delgado JR, 2017 [11]	2014	Peru	AMR	LMIC	NA	NA	Organised population-based	Pap	71	62	19–60
Erku DA, 2017 [12]	2017	Ethiopia	AFR	LMIC	Both	NA	Opportunistic	NA	302	71	21–65
Njuguna E, 2017 [13]	2013	Kenya	AFR	LMIC	NA	KNH CCC	Opportunistic	VIA	387	179	≥18
Wanyenze RK, 2017 [14]	2016	Uganda	AFR	LMIC	NA	NA	Opportunistic	NA	5153	1561	15–49
Koneru A, 2017 [15]	2012	Tanzania	AFR	LMIC	NA	NA	NA	VIA	399	36	≥19
Adibe MO, 2018 [16]	2015	Nigeria	AFR	LMIC	NA	NA	Opportunistic	Pap	447	45	NA
Belglaiaa E, 2018 [17]	2017	Morocco	EMR	LMIC	Both	NA	Organised population-based	Pap	115	15	NA
Nega AD, 2018 [18]	2016	Ethiopia	AFR	LMIC	Both	NA	Opportunistic	Pap / VIA	460	46	≥18
Shiferaw S, 2018 [19]	2016	Kenya	AFR	LMIC	NA	NA	Opportunistic	NA	581	63	21–64
Bulto G, 2019 [20]	2016	Ethiopia	AFR	LMIC	Both	NA	Opportunistic	Pap / VIA	423	9	≥18
de Pokomandy A, 2019 [21]	2014	Canada	AMR	HIC	NA	CHIWOS	Opportunistic	Pap	1189	1138	21–70
Solomon K, 2019 [22]	2018	Ethiopia	AFR	LMIC	NA	NA	Opportunistic	NA	475	118	NA
Tchounga B, 2019 [23]	2017	Côte	AFR	LMIC	Urban	NA	Opportunistic	Pap / VIA	1991	1188	25–55

Study	Year investigated	Country	WHO region	WB region	Study setting	Study name	Type of cervical cancer screening programme	Screening method	No. of participants [a]	No. of lifetime screening	Age range (years)
		d'Ivoire									
Wijayabahu AT, 2019 [24]	2016	USA	AMR	HIC	NA	Florida Cohort	Opportunistic	Pap	190	175	NA
Kohler RE, 2019 [25]	2017	Botswana	AFR	LMIC	Both	NA	Organised population-based	VIA	104	66	≥25
Fitzpatrick M, 2020 [26]	2017	Zimbabwe	AFR	LMIC	Rural	NA	Organised population-based	Pap	145	5	30–65
New-Aaron M, 2020 [27]	2017	Tanzania	AFR	LMIC	Both	NA	Not applicable	VIA	421	49	18–50
Songsiriphan A, 2020 [28]	2019	Thailand	SEAR	LMIC	Both	NA	Organised population-based	Pap	300	269	18–65
Aina IO, 2020 [29]	2014	Eswatini	AFR	LMIC	NA	NA	Organised population-based	VIA	111	34	18–69
Emru K, 2021 [30]	2015	Ethiopia	AFR	LMIC	Urban	NA	Opportunistic	NA	411	105	15–49
Kemper KE, 2021 [31]	2016	Kenya	AFR	LMIC	NA	NA	Opportunistic	Pap / VIA	3007	1671	18–49
Kimondo FC, 2021 [32]	2020	Tanzania	AFR	LMIC	Both	NA	Not applicable	VIA	297	149	18–55
Hopkins KL, 2021 [33]	2018	South Africa	AFR	LMIC	NA	NA	Opportunistic	Pap	57	33	≥18
Musuka G, 2022 [34]	2016	Zimbabwe	AFR	LMIC	Both	ZDHS	Organised population-based	Pap / VIA	1318	311	15–49
Cicconi P, 2022 [35]	2020	UK	EUR	HIC	NA	NA	Organised population-based	Pap	66	61	24–60
Kangethe, JM, 2022 [36]	2020	Kenya	AFR	LMIC	NA	NA	Opportunistic	Pap / VIA	305	136	≥14
Sarah MN, 2022 [37]	2017	Uganda	AFR	LMIC	NA	NA	Opportunistic	NA	205	90	NA
Zhao R, 2022 [38]	2019	China	WPR	LMIC	NA	NA	Organised population-based	Pap / VIA	101	47	21–65
Lin S, 2022 [39]	2019	China	WPR	LMIC	NA	NA	Organised population-based	NA	213	78	20–79

Note: [a] The number of WLWH who were recommended for cervical cancer screening.

Pap = Papanicolaou smear. NA = not available. VIA = Visual Inspection with Acetic Acid.

Study name: CHIWOS = Canadian HIV Women's Sexual and Reproductive Health Cohort Study. KNH CCC = Kenyatta National Hospital Comprehensive Care Centre. PISCIS = Clinical-epidemiological characteristics and antiretroviral treatment trends in a cohort of HIV infected patients. ZDHS = Zimbabwe using 2015-16 Demographic Health Survey.

Region: HIC = High-income country. LMIC = low-income and middle-income country. AFR = WHO African Region. AMR = WHO Region of the Americas. EMR = WHO Eastern Mediterranean Region. EUR = WHO European Region. SEAR = WHO South-East Asia Region. WPR = WHO Western Pacific Region.

Table S7. Basic characteristics of included studies reporting adherence to cervical cancer screening guidelines (n=37)

Study	Year investigated	Country	WHO region	WB region	Study setting	Study name	Type of cervical cancer screening programme	Guideline name	Definition of adherence	Screening method	Screening interval	No. of participants [a]	No. of adherence	Age range (years)
Stein MD, 2001 [40]	1996	USA	AMR	HIC	NA	HCSUS	Opportunistic	USPHS/IDSA guidelines for the prevention of opportunistic infections in persons infected with human immunodeficiency virus. 1999	Pap test every year	Pap	1 year	624	505	≥17
Keiser O, 2006 [41]	2003	Switzerland	EUR	HIC	NA	SHCS	Opportunistic	USPHS/IDSA guidelines for the prevention of opportunistic infections in persons infected with human immunodeficiency virus.1997	Pap test every year	Pap	1 year	1146	1103	NA
Shah S, 2006 [42]	2003	UK	EUR	HIC	NA	NA	Organised population-based	HIV Infection in Maternity Care and Gynecology. 1997	Pap test every year	Pap	1 year	59	31	NA
Oster AM, 2009 [43]	2002	USA	AMR	HIC	NA	Supplement to HIV/AI	Opportunistic	USPHS/IDSA guidelines for the prevention of	Pap test every year	Pap	1 year	2417	1861	≥18

Study	Year investigated	Country	WHO region	WB region	Study setting	Study name	Type of cervical cancer screening programme	Guideline name	Definition of adherence	Screening method	Screening interval	No. of participants [a]	No. of adherence	Age range (years)
						DS Surveillance project		opportunistic infections in persons infected with human immunodeficiency virus. 1995						
Dal Maso L, 2010 [2]	2007	Italy	EUR	HIC	NA	NA	Organised population-based	Consensus guidelines for the management of women with abnormal cervical cancer screening tests. 2006	Pap test every year	Pap	1 year	1002	607	NA
Leece P, 2010 [44]	2004	Canada	AMR	HIC	NA	NA	Opportunistic	Cervical screening: a clinical practice guideline. 2005	Pap test every year	Pap	3 years	218	126	NA
Logan JL, 2010 [45]	2003	USA	AMR	HIC	NA	NA	Opportunistic	Guidelines for prevention and treatment of opportunistic infections in HIV-infected adults and adolescents. 2009	Pap tests twice in the first year after HIV diagnosis	Pap	NA	200	49	≥18
Rahangdale L, 2010	2004	USA	AMR	HIC	NA	NA	Opportunistic	Guidelines for prevention and	Pap test every year	Pap	5 years	69	53	NA

Study	Year investigated	Country	WHO region	WB region	Study setting	Study name	Type of cervical cancer screening programme	Guideline name	Definition of adherence	Screening method	Screening interval	No. of participants [a]	No. of adherence	Age range (years)
[46]								treatment of opportunistic infections in HIV-infected adults and adolescents. 2008						
Tello MA, 2010 [47]	2008	USA	AMR	HIC	Urban	NA	Opportunistic	NA	Pap test every year	Pap	1 year	200	156	23–78
Stuardo V, 2013 [7]	2008	Spain	EUR	HIC	NA	PISCIS	Opportunistic	Catalonian Cervical Cancer Screening Protocol. 2006	Pap test every year	Pap	1 year	415	210	NA
Chen YC, 2013 [48]	2005	China	WPR	LMIC	Urban	NHIRD	Organised population-based	Guidelines for diagnosis and treatment of HIV/AIDS. Taipei: CDC. 2012	Pap test every year	Pap	1 year	1311	193	≥18
Cross SL, 2014 [49]	2010	USA	AMR	HIC	Urban	NA	Opportunistic	Guidelines for prevention and treatment of opportunistic infections in HIV-infected adults and adolescents. 2008	Pap test every year	Pap	1 year	422	222	NA
Fletcher FE, 2014 [50]	2008	USA	AMR	HIC	NA	Parent study	Opportunistic	Guidelines for prevention and treatment of	Pap test every year	Pap	1 year	138	64	NA

Study	Year investigated	Country	WHO region	WB region	Study setting	Study name	Type of cervical cancer screening programme	Guideline name	Definition of adherence	Screening method	Screening interval	No. of participants [a]	No. of adherence	Age range (years)
								opportunistic infections in HIV-infected adults and adolescents. 2009						
Simonsen SE, 2014 [51]	2009	USA	AMR	HIC	NA	NA	Opportunistic	Primary care guidelines for the management of persons infected with human immunodeficiency virus. 2009	Pap test every year	Pap	1 year	192	109	≥18
Dailey GN, 2015 [52]	2007	USA	AMR	HIC	NA	NA	Opportunistic	Sexually transmitted diseases treatment guidelines. 2010	Screening every year	NA	1 year	498	258	NA
Lambert CC, 2015 [53]	2010	USA	AMR	HIC	NA	NA	Opportunistic	Gynecologic care for women with human immunodeficiency virus. 2010	Pap test every year	Pap	1 year	300	132	18–70
Wigfall LT, 2015 [54]	2012	USA	AMR	HIC	Both	NA	Opportunistic	Guidelines for prevention and treatment of opportunistic infections in HIV -	Pap test every year	Pap	1 year	103	84	20–68

Study	Year investigated	Country	WHO region	WB region	Study setting	Study name	Type of cervical cancer screening programme	Guideline name	Definition of adherence	Screening method	Screening interval	No. of participants [a]	No. of adherence	Age range (years)
								infected adults and adolescents. 2009						
Bynum SA, 2016 [55]	2011	USA	AMR	HIC	NA	NA	Opportunistic	Guidelines for prevention and treatment of opportunistic infections in HIV -infected adults and adolescents. 2009	Pap test every year	Pap	1 year	145	118	NA
Frazier EL, 2016 [56]	2010	USA	AMR	HIC	NA	MMP	Opportunistic	Sexually transmitted diseases treatment guidelines. 2010	Pap test every year	Pap	1 year	2270	1771	≥18
Ogunwale AN, 2016 [57]	2012	USA	AMR	HIC	NA	NA	Opportunistic	NA	Pap test every 3 years	Pap	3 years	209	179	21–64
Delgado JR, 2017 [11]	2014	Peru	AMR	LMIC	NA	NA	Organised population-based	Sexually Transmitted Diseases Treatment Guidelines. 2015	Pap test every year	Pap	1 year	71	16	19–60
Tron L, 2017 [58]	2011	France	EUR	HIC	NA	ANRS	Opportunistic	Recommandations du groupe d'experts. Rapport 2013.	Pap test every year	Pap	1 year	740	566	25–65
Wanyenze	2016	Uganda	AFR	LMIC	NA	NA	Opportunistic	NA	Screening	NA	1 year	5153	822	15–49

Study	Year investigated	Country	WHO region	WB region	Study setting	Study name	Type of cervical cancer screening programme	Guideline name	Definition of adherence	Screening method	Screening interval	No. of participants [a]	No. of adherence	Age range (years)
RK, 2017 [14]									every year					
Barnes A, 2018 [59]	2012	USA	AMR	HIC	Urban	PROSPR	Opportunistic	Updated consensus guidelines for the management of abnormal cervical cancer screening tests and cancer precursors. 2012	Pap test every year	Pap	1 year	1490	660	18–64
Burchell AN, 2018 (2008) [60]	2008	Canada	AMR	HIC	Both	NA	Opportunistic	Ontario Cervical Screening Cytology Guidelines Summary. 2012	Pap test every year	Pap	1 year	1484	505	21–69
Burchell AN, 2018 (2009) [60]	2009	Canada	AMR	HIC	Both	NA	Opportunistic	Ontario Cervical Screening Cytology Guidelines Summary. 2012	Pap test every year	Pap	1 year	1534	520	21–69
Burchell AN, 2018 (2010) [60]	2010	Canada	AMR	HIC	Both	NA	Opportunistic	Ontario Cervical Screening Cytology Guidelines Summary. 2012	Pap test every year	Pap	1 year	1603	522	21–69
Burchell AN, 2018	2011	Canada	AMR	HIC	Both	NA	Opportunistic	Ontario Cervical Screening	Pap test every year	Pap	1 year	1678	556	21–69

Study	Year investigated	Country	WHO region	WB region	Study setting	Study name	Type of cervical cancer screening programme	Guideline name	Definition of adherence	Screening method	Screening interval	No. of participants [a]	No. of adherence	Age range (years)
(2011) [60]								Cytology Guidelines Summary. 2012						
Burchell AN, 2018 (2012) [60]	2012	Canada	AMR	HIC	Both	NA	Opportunistic	Ontario Cervical Screening Cytology Guidelines Summary. 2012	Pap test every year	Pap	1 year	1772	565	21–69
Burchell AN, 2018 (2013) [60]	2013	Canada	AMR	HIC	Both	NA	Opportunistic	Ontario Cervical Screening Cytology Guidelines Summary. 2012	Pap test every year	Pap	1 year	1829	474	21–69
Mohammed DY, 2018 [61]	2016	USA	AMR	HIC	Urban	NA	Opportunistic	Guidelines for the Prevention and Treatment of Opportunistic Infections in HIV-Infected Adults and Adolescents. 2015	Pap test every year	Pap	1 year	360	270	≥18
Assefa AA, 2019 [62]	2019	Ethiopia	AFR	LMIC	NA	NA	Opportunistic	Guideline for Cervical Cancer Prevention and Control in	Screening every 5 years	VIA	5 years	342	137	≥18

Study	Year investigated	Country	WHO region	WB region	Study setting	Study name	Type of cervical cancer screening programme	Guideline name	Definition of adherence	Screening method	Screening interval	No. of participants [a]	No. of adherence	Age range (years)
								Ethiopia. Addis 582 Ababa. 2015						
de Pokomandy A, 2019 [21]	2014	Canada	AMR	HIC	NA	CHIWOS	Opportunistic	Ontario cervical screening guidelines summary. 2016	Pap test every year	Pap	1 year	1189	815	21–70
Short WR, 2019 [63]	2014	USA	AMR	HIC	NA	MMP	Opportunistic	Primary care guidelines for the management of persons infected with HIV. 2013	Pap tests every 2 years	Pap	2 years	2766	1248	≥18
Solomon K, 2019 [22]	2018	Ethiopia	AFR	LMIC	NA	NA	Opportunistic	Guideline for cervical cancer prevention and control in Ethiopia. 2015	Screening every year	NA	1 year	475	98	NA
Wijayabahu AT, 2019 [24]	2016	USA	AMR	HIC	NA	Florida Cohort	Opportunistic	NA	Pap test every 3 years	Pap	3 years	190	160	NA
Songsiriphan A, 2020 [28]	2019	Thailand	SEAR	LMIC	Both	NA	Organised population-based	Thailand national guidelines on HIV/AIDS treatment and prevention. 2017	Annual screening if CD4 levels are <500 cell/mm3 and	Pap	1/2/3 years	300	214	18–65

Study	Year investigated	Country	WHO region	WB region	Study setting	Study name	Type of cervical cancer screening programme	Guideline name	Definition of adherence	Screening method	Screening interval	No. of participants [a]	No. of adherence	Age range (years)
									cytological testing every two years and co-testing every 3 years if CD4 levels were >=500 cell/mm3					
Kemper KE, 2021 [31]	2016	Kenya	AFR	LMIC	NA	NA	Organised population-based	National Guidelines for Cancer Management: Kenya. 2013	Screening every year	Pap or VIA	1 year	3007	804	18–49
Kimondo FC, 2021 [32]	2020	Tanzania	AFR	LMIC	Both	NA	Not applicable	National guidelines for the management of HIV and AIDS. 2017	VIA every year	VIA	1 year	297	96	18–55
Cicconi P, 2022 [35]	2020	UK	EUR	HIC	Both	NA	Organised population-based	BHIVA guidelines on the routine investigation and monitoring of	Pap test every year	Pap	1 year	66	50	24–60

Study	Year investigated	Country	WHO region	WB region	Study setting	Study name	Type of cervical cancer screening programme	Guideline name	Definition of adherence	Screening method	Screening interval	No. of participants [a]	No. of adherence	Age range (years)
								HIV-1-positive adults. 2019						
Sarah MN, 2022 [37]	2017	Uganda	AFR	LMIC	Both	NA	Opportunistic	Sexually transmitted diseases treatment guidelines. 2015	Screening every year	NA	1 year	205	33	NA
Lin S, 2022 [39]	2019	China	WPR	LMIC	Both	NA	Organised population-based	Comprehensive prevention and control guidelines for cervical cancer. 2017	Screening every year	NA	1 year	213	35	20–79

Note: [a] The number of WLWH who were recommended for cervical cancer screening in each study.

Pap = Papanicolaou smear. NA = not available. VIA = Visual Inspection with Acetic Acid.

Study name: ANRS: Agence Nationale de Recherche sur le Sida et les Hépatites Virales. CHIWOS = Canadian HIV Women's Sexual and Reproductive Health Cohort Study. HCSUS=HIV Cost and Service Utilization Study. NHIRD = National Health Insurance Research Database. MMP = Medical Monitoring Project. PISCIS = Clinical-epidemiological characteristics and antiretroviral treatment trends in a cohort of HIV infected patients. PROSPR = Population-Based Optimizing Screening through Personalized Regimens. SHCS = Swiss HIV Cohort Study.

Guideline name: BHIVA = British HIV Association. IDSA = Infectious Diseases Society of America. USPHS=U.S. Public Health Service.

Region: HIC = High-income country. LMIC = low-income and middle-income country. AFR = WHO African Region. AMR = WHO Region of the Americas. EUR = WHO European Region. SEAR = WHO South-East Asia Region.

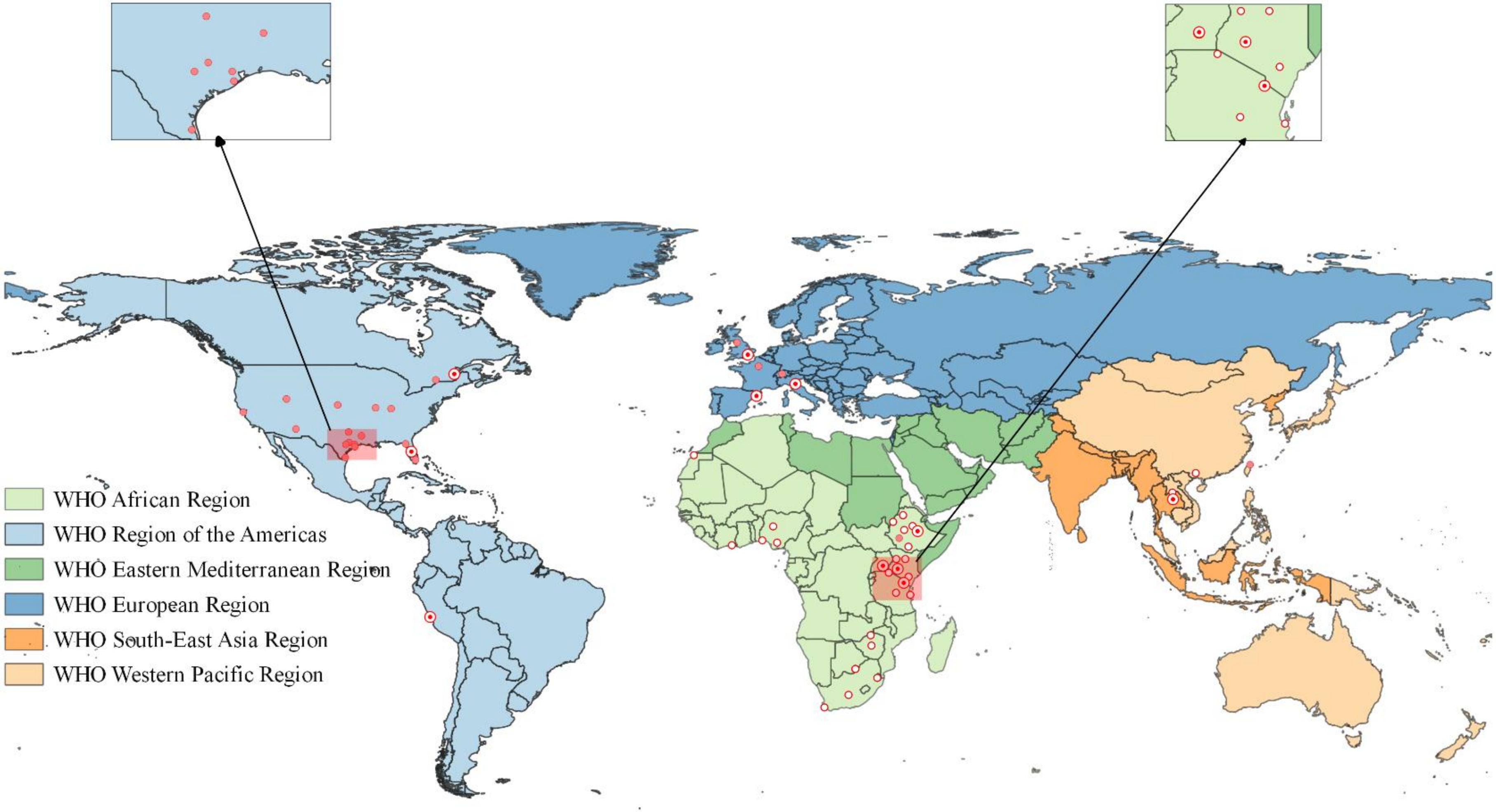

Figure S1. Geographic distribution of the included studies

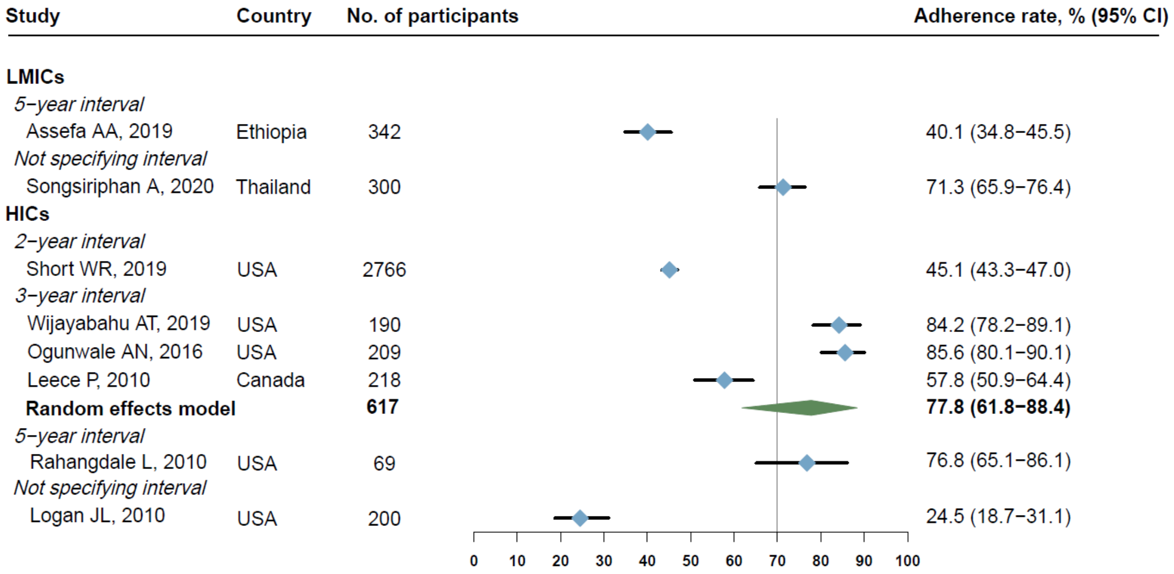

Figure S2. Adherence rates of cervical cancer screening with 2-year, 3-year, 5-year interval and not specifying interval

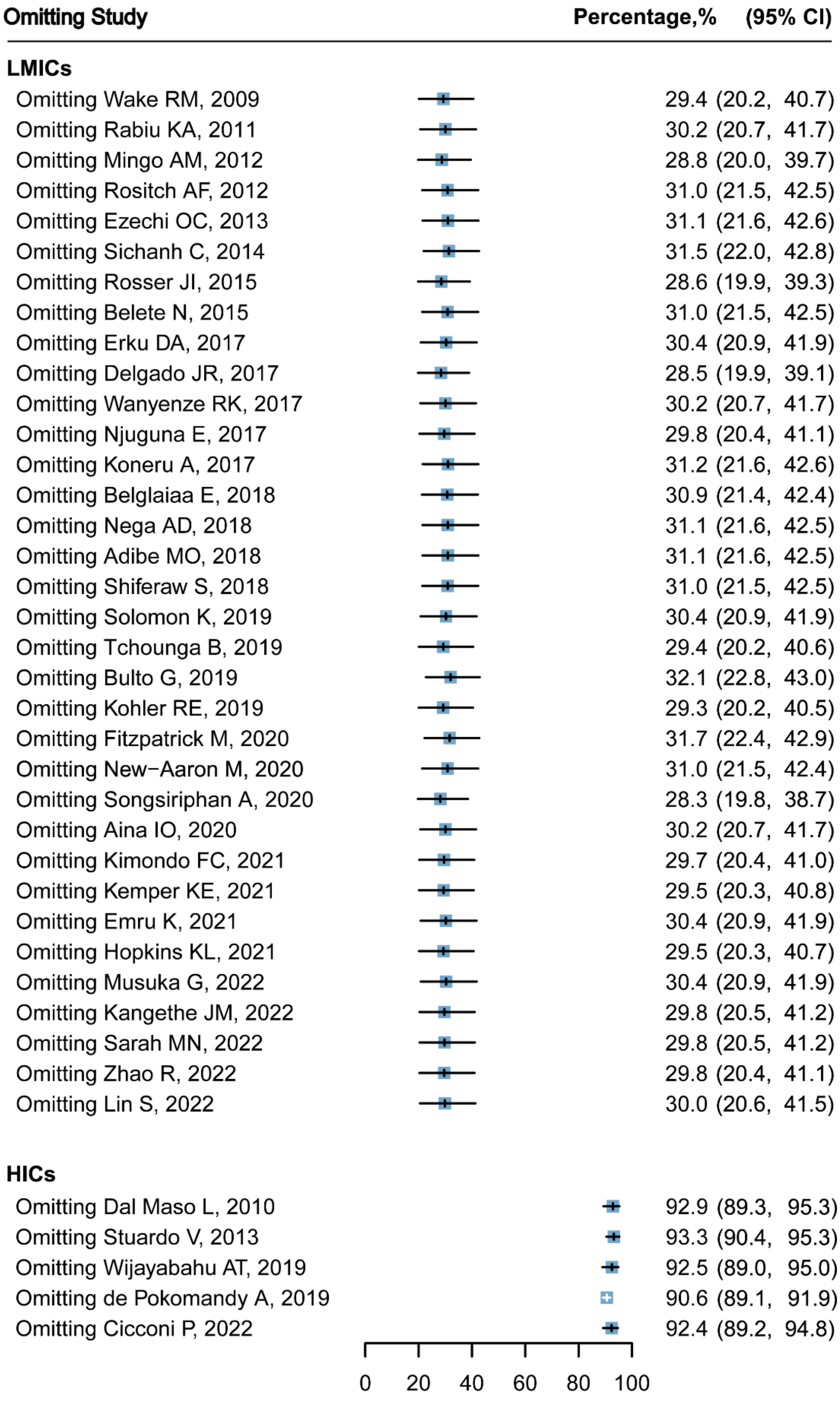

Figure S3. Level-one-out sensitivity analysis of the influence of single study on the pooled cervical cancer screening lifetime prevalence

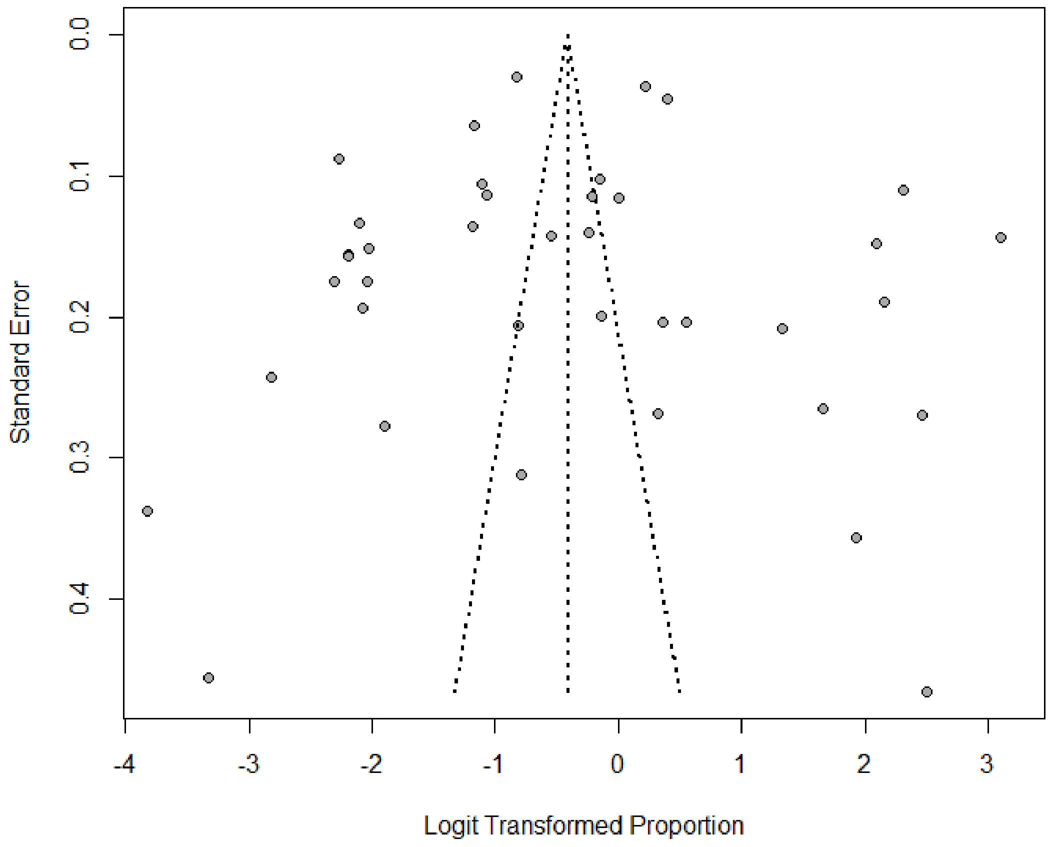

Figure S4. Publication bias of studies on the cervical cancer screening lifetime prevalence

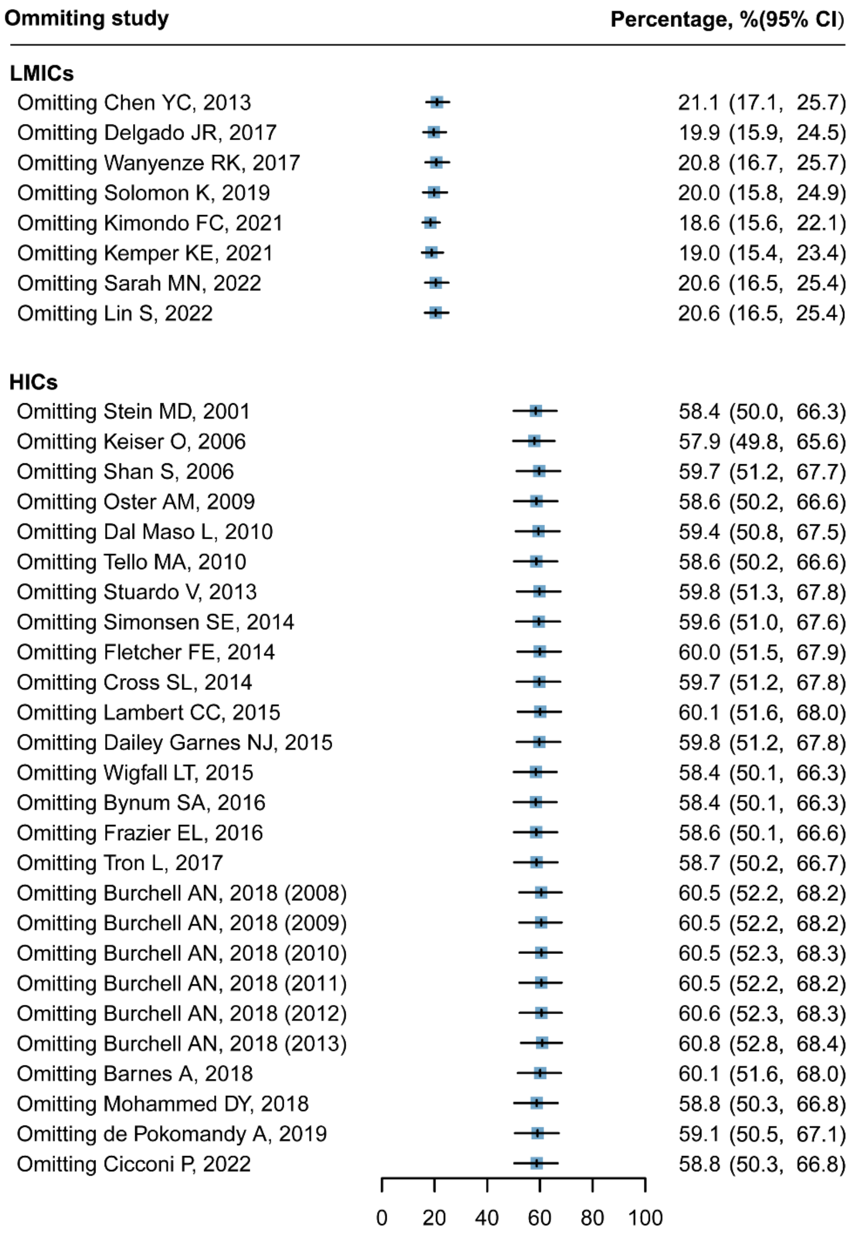

Figure S5. Level-one-out sensitivity analysis of the influence of single study on the pooled cervical cancer screening adherence rate

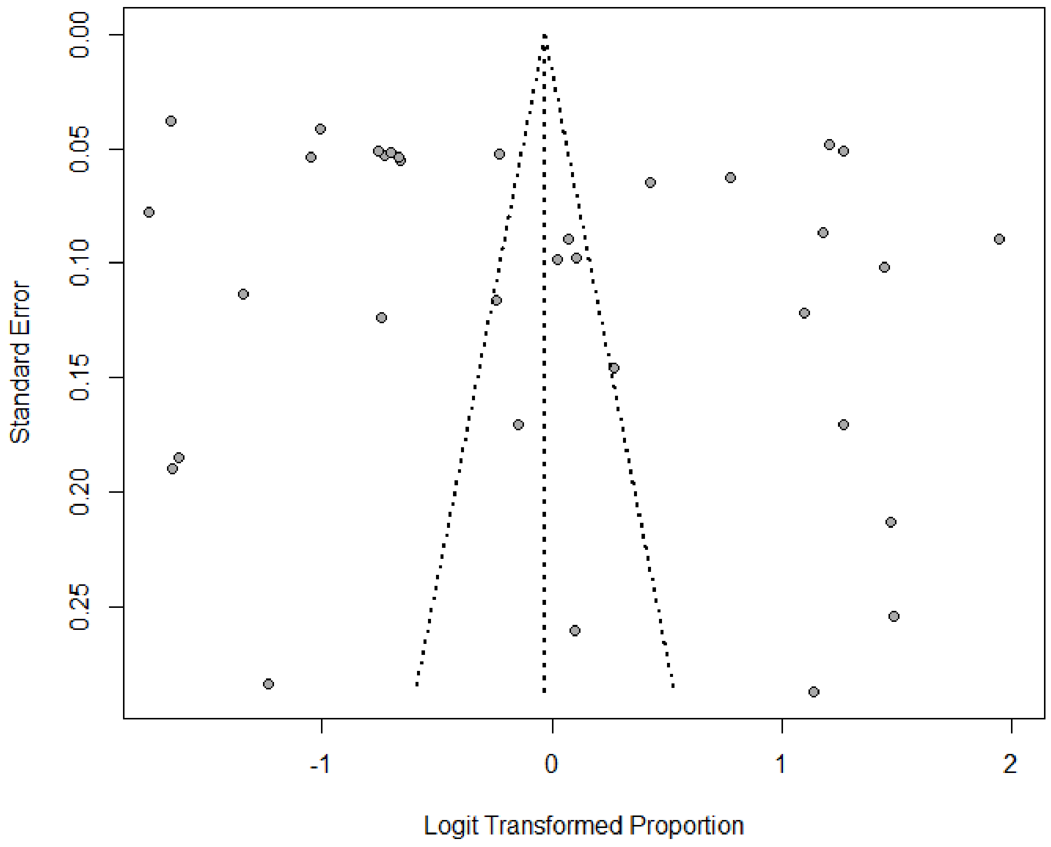

Figure S6. Publication bias of studies on the cervical cancer screening adherence

Appendix. PRISMA checklist

Section and Topic	Item #	Checklist item	Location where item is reported
**TITLE**			
Title	1	Identify the report as a systematic review.	1
**ABSTRACT**			
Abstract	2	See the PRISMA 2020 for Abstracts checklist.	2
**INTRODUCTION**			
Rationale	3	Describe the rationale for the review in the context of existing knowledge.	3
Objectives	4	Provide an explicit statement of the objective(s) or question(s) the review addresses.	3-4
**METHODS**			
Eligibility criteria	5	Specify the inclusion and exclusion criteria for the review and how studies were grouped for the syntheses.	4-5
Information sources	6	Specify all databases, registers, websites, organisations, reference lists and other sources searched or consulted to identify studies. Specify the date when each source was last searched or consulted.	4
Search strategy	7	Present the full search strategies for all databases, registers and websites, including any filters and limits used.	4, Table S1
Selection process	8	Specify the methods used to decide whether a study met the inclusion criteria of the review, including how many reviewers screened each record and each report retrieved, whether they worked independently, and if applicable, details of automation tools used in the process.	4-5
Data collection process	9	Specify the methods used to collect data from reports, including how many reviewers collected data from each report, whether they worked independently, any processes for obtaining or confirming data from study investigators, and if applicable, details of automation tools used in the process.	5-6
Data items	10a	List and define all outcomes for which data were sought. Specify whether all results that were compatible with each outcome domain in each study were sought (e.g. for all measures, time points, analyses), and if not, the methods used to decide which results to collect.	5-6
	10b	List and define all other variables for which data were sought (e.g. participant and intervention characteristics, funding sources). Describe any assumptions made about any missing or unclear information.	5-6
Study risk of bias assessment	11	Specify the methods used to assess risk of bias in the included studies, including details of the tool(s) used, how many reviewers assessed each study and whether they worked independently, and if applicable, details of automation tools used in the process.	5, Table S2

Section and Topic	Item #	Checklist item	Location where item is reported
Effect measures	12	Specify for each outcome the effect measure(s) (e.g. risk ratio, mean difference) used in the synthesis or presentation of results.	6
Synthesis methods	13a	Describe the processes used to decide which studies were eligible for each synthesis (e.g. tabulating the study intervention characteristics and comparing against the planned groups for each synthesis (item #5)).	6
	13b	Describe any methods required to prepare the data for presentation or synthesis, such as handling of missing summary statistics, or data conversions.	6
	13c	Describe any methods used to tabulate or visually display results of individual studies and syntheses.	6
	13d	Describe any methods used to synthesize results and provide a rationale for the choice(s). If meta-analysis was performed, describe the model(s), method(s) to identify the presence and extent of statistical heterogeneity, and software package(s) used.	6
	13e	Describe any methods used to explore possible causes of heterogeneity among study results (e.g. subgroup analysis, meta-regression).	6
	13f	Describe any sensitivity analyses conducted to assess robustness of the synthesized results.	6
Reporting bias assessment	14	Describe any methods used to assess risk of bias due to missing results in a synthesis (arising from reporting biases).	6
Certainty assessment	15	Describe any methods used to assess certainty (or confidence) in the body of evidence for an outcome.	6
**RESULTS**			
Study selection	16a	Describe the results of the search and selection process, from the number of records identified in the search to the number of studies included in the review, ideally using a flow diagram.	7, Figure 1
	16b	Cite studies that might appear to meet the inclusion criteria, but which were excluded, and explain why they were excluded.	7, Figure 1
Study characteristics	17	Cite each included study and present its characteristics.	7, Table S6&S7, Figure S1
Risk of bias in studies	18	Present assessments of risk of bias for each included study.	Table S5
Results of individual studies	19	For all outcomes, present, for each study: (a) summary statistics for each group (where appropriate) and (b) an effect estimate and its precision (e.g. confidence/credible interval), ideally using structured tables or plots.	8-9, Figure 2
Results of	20a	For each synthesis, briefly summarise the characteristics and risk of bias among contributing studies.	8-9

Section and Topic	Item #	Checklist item	Location where item is reported
syntheses	20b	Present results of all statistical syntheses conducted. If meta-analysis was done, present for each the summary estimate and its precision (e.g. confidence/credible interval) and measures of statistical heterogeneity. If comparing groups, describe the direction of the effect.	8-9, Table 1&2
	20c	Present results of all investigations of possible causes of heterogeneity among study results.	Table 1&2
	20d	Present results of all sensitivity analyses conducted to assess the robustness of the synthesized results.	Figure S3&S5
Reporting biases	21	Present assessments of risk of bias due to missing results (arising from reporting biases) for each synthesis assessed.	Figure S4&S6
Certainty of evidence	22	Present assessments of certainty (or confidence) in the body of evidence for each outcome assessed.	Figure 2, Table 1&2
**DISCUSSION**			
Discussion	23a	Provide a general interpretation of the results in the context of other evidence.	9-11
	23b	Discuss any limitations of the evidence included in the review.	11
	23c	Discuss any limitations of the review processes used.	11
	23d	Discuss implications of the results for practice, policy, and future research.	12
**OTHER INFORMATION**			
Registration and protocol	24a	Provide registration information for the review, including register name and registration number, or state that the review was not registered.	4
	24b	Indicate where the review protocol can be accessed, or state that a protocol was not prepared.	4
	24c	Describe and explain any amendments to information provided at registration or in the protocol.	NA
Support	25	Describe sources of financial or non-financial support for the review, and the role of the funders or sponsors in the review.	13
Competing interests	26	Declare any competing interests of review authors.	13
Availability of data, code and other materials	27	Report which of the following are publicly available and where they can be found: template data collection forms; data extracted from included studies; data used for all analyses; analytic code; any other materials used in the review.	13

References

1. Wake RM, Rebe K, Burch VC. Patient perception of cervical screening among women living with human immuno-deficiency virus infection attending an antiretroviral therapy clinic in urban South Africa. J Obstet Gynaecol. 2009;29(1):44-48.
2. Dal Maso L, Franceschi S, Lise M, De' Bianchi PS, Polesel J, Ghinelli F, et al. Self-reported history of Pap-smear in HIV-positive women in Northern Italy: A cross-sectional study. BMC Cancer. 2010;10.
3. Rabiu KA, Akinbami AA, Adewunmi AA, Akinola OI, Wright KO. The need to incorporate routine cervical cancer counselling and screening in the management of HIV positive women in nigeria. Asian Pac J Cancer Prev. 2011;12(5):1211-14.
4. Mingo AM, Panozzo CA, Diangi YT, Smith JS, Steenhoff AP, Ramogola-Masire D, et al. Cervical cancer awareness and screening in Botswana. Int J Gynecol Cancer. 2012;22(4):638-44.
5. Rositch AF, Gatuguta A, Choi RY, Guthrie BL, Mackelprang RD, Bosire R, et al. Knowledge and acceptability of pap smears, self-sampling and HPV vaccination among adult women in Kenya. PloS One. 2012;7(7):e40766.
6. Ezechi OC, Gab-Okafor CV, Ostergren PO, Odberg PK. Willingness and acceptability of cervical cancer screening among HIV positive Nigerian women. BMC Public Health. 2013;13:46.
7. Stuardo V, Agustí C, Casabona J. Low prevalence of cervical cancer screening among HIV-positive women in Catalonia (Spain). ID - 20133280014. Journal of AIDS and Clinical Research. 2013;(No.Suppl. 3):4.
8. Sichanh C, Quet F, Chanthavilay P, Diendere J, Latthaphasavang V, Longuet C, et al. Knowledge, awareness and attitudes about cervical cancer among women attending or not an HIV treatment center in Lao PDR. BMC Cancer. 2014;14(1).
9. Belete N, Tsige Y, Mellie H. Willingness and acceptability of cervical cancer screening among women living with HIV/AIDS in Addis Ababa, Ethiopia: A cross sectional study. Gynecol Oncol Res Pract. 2015;2:6.
10. Rosser JI, Njoroge B, Huchko MJ. Cervical cancer screening knowledge and behavior among women attending an urban HIV clinic in western kenya. J Cancer Educ. 2015;30(3):567-72.
11. Delgado JR, Menacho L, Segura ER, Roman F, Cabello R. Cervical cancer screening practices, knowledge of screening and risk, and highly active antiretroviral therapy adherence among women living with human immunodeficiency virus in Lima, Peru. International Journal of STD and AIDS. 2017;28(3):290-93.
12. Erku DA, Netere AK, Mersha AG, Abebe SA, Mekuria AB, Belachew SA. Comprehensive knowledge and uptake of cervical cancer screening is low among women living with HIV/AIDS in Northwest Ethiopia. Gynecologic Oncology Research and Practice. 2017;4(1).
13. Njuguna E, Ilovi S, Muiruri P, Mutai K, Kinuthia J, Njoroge P. Factors influencing cervical cancer screening in a Kenyan Health Facility: A mixed qualitative and quantitative study. International Journal of Reproduction, Contraception, Obstetrics and Gynecology. 2017;6(4):1180-85.
14. Wanyenze RK, Bwanika JB, Beyeza-Kashesya J, Mugerwa S, Arinaitwe J, Matovu JKB, et al. Uptake and correlates of cervical cancer screening among HIV-infected women attending HIV care in Uganda. Global Health Action. 2017;10(13803611).
15. Koneru A, Jolly PE, Blakemore S, Mccree R, Lisovicz NF, Aris EA, et al. Acceptance of peer navigators to reduce barriers to cervical cancer screening and treatment among women with HIV

infection in Tanzania. Int J Gynaecol Obstet. 2017;138(1):53-61.
16. Adibe MO, Aluh DO. Awareness, knowledge and attitudes towards cervical cancer amongst HIV-Positive women receiving care in a tertiary hospital in nigeria. J Cancer Educ. 2018;33(6):1189-94.
17. Belglaiaa E, Souho T, Badaoui L, Segondy M, Prétet JL, Guenat D, et al. Awareness of cervical cancer among women attending an HIV treatment centre: A cross-sectional study from Morocco. BMJ Open. 2018;8(8).
18. Nega AD, Woldetsadik MA, Gelagay AA. Low uptake of cervical cancer screening among HIV positive women in Gondar University referral hospital, Northwest Ethiopia: Cross-sectional study design. BMC Women's Health. 2018;18(1).
19. Shiferaw S, Addissie A, Gizaw M, Hirpa S, Ayele W, Getachew S, et al. Knowledge about cervical cancer and barriers toward cervical cancer screening among HIV-positive women attending public health centers in Addis Ababa city, Ethiopia. Cancer Med. 2018;7(3):903-12.
20. Bulto G, Demmissie D, Daka K. Knowledge about cervical cancer, screening practices, and associated factors among women living with HIV in public hospitals of west shoa zone, central ethiopia. J Women's Health Care. 2019;(8).
21. de Pokomandy A, Burchell AN, Salters K, Ding E, O'Brien N, Bakombo DM, et al. Cervical cancer screening among women living with HIV: A cross-sectional study using the baseline questionnaire data from the Canadian HIV Women's Sexual and Reproductive Health Cohort Study (CHIWOS). CMAJ Open. 2019;7(2):E217-26.
22. Solomon K, Tamire M, Kaba M. Predictors of cervical cancer screening practice among HIV positive women attending adult anti-retroviral treatment clinics in Bishoftu town, Ethiopia: The application of a health belief model. Bmc Cancer. 2019;19(1).
23. Tchounga B, Boni SP, Koffi JJ, Horo AG, Tanon A, Messou E, et al. Cervical cancer screening uptake and correlates among HIV-infected women: A cross-sectional survey in Côte d'Ivoire, West Africa. BMJ Open. 2019;9(8).
24. Wijayabahu AT, Zhou Z, Cook RL, Brumback B, Ennis N, Yaghjyan L. Healthy behavioral choices and cancer screening in persons living with HIV/AIDS are different by sex and years since HIV diagnosis. Cancer Causes and Control. 2019;30(3):281-90.
25. Kohler RE, Elliott T, Monare B, Moshashane N, Ramontshonyana K, Chatterjee P, et al. HPV self-sampling acceptability and preferences among women living with HIV in Botswana. Int J Gynaecol Obstet. 2019;147(3):332-38.
26. Fitzpatrick M, Pathipati MP, Mccarty K, Rosenthal A, Katzenstein D, Chirenje ZM, et al. Knowledge, attitudes, and practices of cervical Cancer screening among HIV-positive and HIV-negative women participating in human papillomavirus screening in rural Zimbabwe. BMC Women's Health. 2020;20(1).
27. New-Aaron M, Meza JL, Goedert MH, Kibusi SM, Mkhoi ML, Mayengo CD, et al. Cervical cancer screening among women receiving antiretroviral therapy in a Resource-Limited environment. Asian Pac J Cancer Prev. 2020;21(7):2035-45.
28. Songsiriphan A, Salang L, Somboonpha W, Eamudomkarn N, Nhokaew W, Kuchaisit C, et al. Knowledge, attitudes, and practices regarding cervical cancer screening among HIV-infected women at srinagarind hospital: A Cross-Sectional study. Asian Pac J Cancer Prev. 2020;21(10):2979-86.
29. Aina IO, Raul SM, Padilla LA, Mthethwa-Hleta S, Preko PO, Jolly PE. Sociodemographic

factors, health seeking behaviors, reproductive history, and knowledge of cervical screening among women in Swaziland. Infect Agent Cancer. 2020;15:16.
30. Emru K, Abebaw T, Abera A. Role of awareness on cervical cancer screening uptake among HIV positive women in Addis Ababa, Ethiopia: A cross-sectional study. Women's health (Lond). 2021;17:292493137.
31. Kemper KE, Mcgrath CJ, Eckert LO, Kinuthia J, Singa B, Langat A, et al. Correlates of cervical cancer screening among women living with HIV in Kenya: A cross-sectional study. International Journal of Gynecology and Obstetrics. 2021.
32. Kimondo FC, Kajoka HD, Mwantake MR, Amour C, Mboya IB. Knowledge, attitude, and practice of cervical cancer screening among women living with HIV in the Kilimanjaro region, northern Tanzania. Cancer Reports. 2021.
33. Hopkins KL, Jaffer M, Hlongwane KE, Otwombe K, Dietrich J, Cheyip M, et al. Assessing national cervical cancer screening guidelines: Results from an HIV esting clinic also screening for cervical cancer and HPV in Soweto, South Africa. PloS One. 2021;16(7):e255124.
34. Musuka G, Mukandavire Z, Murewanhema G, Cuadros D, Mutenherwa F, Chingombe I, et al. HIV status, knowledge and prevention of cervical cancer amongst adolescent girls and women: A secondary data analysis. Pan Afr Med J. 2022;41:262.
35. Cicconi P, Wells C, Mccarthy B, Wareing S, Andersson MI, Fox J, et al. Re-valuation of annual cytology using HPV self-sampling to upgrade prevention (REACH UP): A feasibility study in women living with HIV in the UK. HIV Med. 2022;23(4):390-96.
36. Kangethe JM, Monroe-Wise A, Muiruri PN, Komu JG, Mutai KK, Nzivo MM, et al. Utilisation of cervical cancer screening among women living with HIV at Kenya's national referral hospital. South Afr J HIV Med. 2022;23(1):1353.
37. Sarah MN, Olwit C, Kaggwa MM, Nabirye RC, Ngabirano TD. Cervical cancer screening among HIV-positive women in urban Uganda: A cross sectional study. BMC Womens Health. 2022;22(1):148.
38. Zhao R, Liang S, Teoh D, Fei Y, Pang X, Kulasingam S. Correction: A comprehensive cross-sectional survey to identify barriers and facilitators of cervical cancer screening in women with HIV in Guangxi, China. Infect Agent Cancer. 2022;17(1):21.
39. Lin S, Chen WT, Gu C, Cheng HL, Wang H, Tang S. Knowledge, perception of HIV symptom severity and cervical cancer screening behaviour among women living with HIV in China. Eur J Cancer Care (Engl). 2022;31(2):e13542.
40. Stein MD, Cunningham WE, Nakazono T, Turner BJ, Andersen RM, Bozzette SA, et al. Screening for cervical cancer in HIV-infected women receiving care in the United States. Journal of Acquired Immune Deficiency Syndromes. 2001;27(5):463-66.
41. Keiser O, Martinez DTB, Wunder D, Chapuis-Taillard C, Zellweger C, Zinkernagel AS, et al. Frequency of gynecologic follow-up and cervical cancer screening in the Swiss HIV cohort study. J Acquir Immune Defic Syndr. 2006;43(5):550-55.
42. Shah S, Montgomery H, Smith C, Madge S, Walker P, Evans H, et al. Cervical screening in HIV-positive women: Characteristics of those who default and attitudes towards screening. HIV Med. 2006;7(1):46-52.
43. Oster AM, Sullivan PS, Blair JM. Prevalence of cervical cancer screening of HIV-infected women in the United States. J Acquir Immune Defic Syndr. 2009;51(4):430-36.
44. Leece P, Kendall C, Touchie C, Pottie K, Angel JB, Jaffey J. Cervical cancer screening among

HIV-positive women. Retrospective cohort study from a tertiary care HIV clinic. Can Fam Physician. 2010;56(12):e425-31.
45. Logan JL, Khambaty MQ, D'Souza KM, Menezes LJ. Cervical cancer screening among HIV-infected women in a health department setting. Aids Patient Care St. 2010;24(8):471-75.
46. Rahangdale L, Sarnquist C, Yavari A, Blumenthal P, Israelski D. Frequency of cervical cancer and breast cancer screening in HIV-infected women in a county-based HIV clinic in the Western United States. Journal of Women's Health. 2010;19(4):709-12.
47. Tello MA, Jenckes M, Gaver J, Anderson JR, Moore RD, Chander G. Barriers to recommended gynecologic care in an urban united states HIV clinic. J Womens Health. 2010;19(8):1511-18.
48. Chen YC, Liu HY, Li CY, Lee NY, Ko WC, Chou CY, et al. Low Papanicolaou smear screening rate of women with HIV infection: A nationwide population-based study in Taiwan, 2000-2010. J Womens Health (Larchmt). 2013;22(12):1016-22.
49. Cross SL, Suharwardy SH, Bodavula P, Schechtman K, Overton ET, Onen NF, et al. Improving cervical cancer screening rates in an urban HIV clinic. Aids Care. 2014;26(9):1186-93.
50. Fletcher FE, Vidrine DJ, Tami-Maury I, Danysh HE, King RM, Buchberg M, et al. Cervical Cancer Screening Adherence among HIV-Positive Female Smokers from a Comprehensive HIV Clinic. AIDs Behav. 2014;18(3):544-54.
51. Simonsen SE, Kepka D, Thompson J, Warner EL, Snyder M, Ries KM. Preventive health care among HIV positive women in a Utah HIV/AIDS clinic: A retrospective cohort study. BMC Women's Health. 2014;14(1).
52. Dailey GN, D'Souza G, Chiao E. Number of primary care visits associated with screening for cervical dysplasia among women with HIV infection in harris county, texas, united states of america. HIV Adv Res Dev. 2015;1(2).
53. Lambert CC, Chandler R, Mcmillan S, Kromrey J, Johnson-Mallard V, Kurtyka D. Pap test adherence, cervical cancer perceptions, and HPV knowledge among HIV-Infected women in a community health setting. J Assoc Nurses AIDs Care. 2015;26(3):271-80.
54. Wigfall LT, Bynum SA, Brandt HM, Friedman DB, Bond SM, Lazenby GB, et al. Cervical cancer prevention knowledge and abnormal pap test experiences among women living with HIV/AIDS. J Cancer Educ. 2015;30(2):213-19.
55. Bynum SA, Wigfall LT, Brandt HM, Julious CH, Glover SH, Hebert JR. Social and structural determinants of cervical health among women engaged in HIV care. AIDs Behav. 2016;20(9):2101-09.
56. Frazier EL, Sutton MY, Tie Y, Mcnaghten AD, Blair JM, Skarbinski J. Screening for cervical cancer and sexually transmitted diseases among HIV-Infected women. J Womens Health (Larchmt). 2016;25(2):124-32.
57. Ogunwale AN, Coleman MA, Sangi-Haghpeykar H, Valverde I, Montealegre J, Jibaja-Weiss M, et al. Assessment of factors impacting cervical cancer screening among low-income women living with HIV-AIDS. AIDS Care - Psychological and Socio-Medical Aspects of AIDS/HIV. 2016;28(4):491-94.
58. Tron L, Lert F, Spire B, Dray-Spira R, Allègre T, Mours P, et al. Levels and determinants of breast and cervical cancer screening uptake in HIV-infected women compared with the general population in France. HIV Med. 2017;18(3):181-95.
59. Barnes A, Betts AC, Borton EK, Sanders JM, Pruitt SL, Werner C, et al. Cervical cancer screening among HIV-infected women in an urban, United States safety-net healthcare system. Aids.

2018;32(13):1861-70.

60. Burchell AN, Kendall CE, Cheng SY, Lofters A, Cotterchio M, Bayoumi AM, et al. Cervical cancer screening uptake among HIV-positive women in Ontario, Canada: A population-based retrospective cohort study. Prev Med. 2018;107:14-20.

61. Mohammed DY, Shukla P, Babayants Y, Sison R, Slim J. Increased proportions of HIV-infected women met cervical cancer screening guideline in 2016. Int J Womens Health. 2018;10:83-87.

62. Assefa AA, Astawesegn FH, Eshetu B. Cervical cancer screening service utilization and associated factors among HIV positive women attending adult ART clinic in public health facilities, Hawassa town, Ethiopia: A cross-sectional study. Bmc Health Serv Res. 2019;19(8471).

63. Short WR, Sutton MY, Luo Q, Frazier EL. Use of recommended preventive health care services and variations in HIV care among women with HIV in the United States, 2013-2014: Opportunities for expanded partnerships in support of ending the HIV epidemic. Journal of Acquired Immune Deficiency Syndromes. 2019;82(3):234-44.
